# Technology-Supported Physical Activity and Its Potential as a Tool to Promote Young Women’s Physical Activity and Physical Literacy: Systematic Review

**DOI:** 10.2196/52302

**Published:** 2024-10-18

**Authors:** Kimberley Watson-Mackie, Lauren Arundell, Natalie Lander, Fiona H McKay, Alethea Jerebine, Fotini Venetsanou, Lisa M Barnett

**Affiliations:** 1 School of Health and Social Development, Faculty of Health Deakin University Burwood Australia; 2 Institute for Physical Activity and Nutrition, Faculty of Health School of Exercise and Nutrition Sciences Deakin University Burwood Australia; 3 Institute for Health Transformation, Faculty of Health School of Health and Social Development Deakin University Burwood Australia; 4 School of Physical Education and Sport Science National and Kapodistrian University of Athens Athens Greece; 5 Institute for Physical Activity and Nutrition, Faculty of Health School of Health and Social Development Deakin University Burwood Australia

**Keywords:** physical literacy, young women, girls, adolescents, technology, mobile, digital, fitness, exercise, physical activity, technology-supported, systematic review, digital health, eHealth, health informatics, mobile phone

## Abstract

**Background:**

Despite the known benefits of physical activity (PA), rates of engagement in PA remain low globally. Low engagement in PA among young women can impact their health. Technology-supported PA may increase PA and physical literacy (PL; skills that can support PA) among young women.

**Objective:**

This systematic review aims to investigate the (1) associations between technology-supported PA and PA levels, (2) associations between technology-supported PA and PL levels, and (3) types of technology-supported PA that are associated with higher levels of PA engagement among young women aged 13 to 24 years. This age range was chosen as it includes two transitional periods characterized by decreases in PA.

**Methods:**

We searched 6 databases: Applied Science and Technology Source, Education Source, Embase, MEDLINE Complete, Global Health, and SPORTDiscus. Eligible studies were original research published in English between January 1, 2010, and April 24, 2024; focused on young women; and involving either technology-supported PA interventions or research exploring the correlation between technology and PA and PL. The findings of the review were presented descriptively. Study quality was assessed using the JBI Critical Appraisal tools. There were no deviations from the registered protocol.

**Results:**

In total, 23 (0.1%) studies (10,233 participants) from 23,609 records were included: randomized controlled trials (n=9, 39%), nonrandomized or retrospective observational studies (n=9, 39%), and cross-sectional studies (n=5, 22%). Of the 23 studies, 12 (52%) focused on young adults (aged ≥19 y), 9 (39%) involved adolescents (aged <19 y), and 2 (9%) focused on both groups. Nine studies (39%) were theory based. Common types of technology-supported PA were interactive websites or social media platforms (10/23, 43%), wearable fitness trackers (4/23, 17%), and mobile apps (4/23, 17%). PA or PL were predominantly self-reported (18/23, 78%). A total of 53 PA outcomes were measured: 36% (19/53) reported a positive impact on PA from mobile apps (9/15; 60% of analyses), interactive websites or social media platforms (8/27; 30% of analyses) and wearable fitness trackers (2/11; 18% of analyses). The impact on PL was weak (2/7; 29% of analyses). Eight studies (35%) were rated high, 7 (30%) medium, and 8 (35%) low in quality.

**Conclusions:**

There was limited evidence that technology-supported PA improved young women’s PA or PL. The evidence was limited by poor study quality and a lack of theoretical frameworks. In addition, little information was provided on the designs of the technology used. Future interventions seeking to improve young women’s PA and PL should focus on the development of mobile apps underpinned by behavior change theory and addressing whole domains of PL rather than specific elements. Given that technology continues to rapidly advance, further studies are needed to demonstrate the impact of technology-supported PA in improving PA and PL among young women.

## Introduction

### Background

Physical activity (PA) is considered one of the most effective ways to maintain good health across the lifespan [1,2]. The World Health Organization (WHO) PA guidelines recommend at least 60 minutes of PA per day for those aged 5 to 17 years and 150 to 300 minutes per week for those aged 18 to 64 years [3]. Meeting the WHO PA guidelines helps individuals maintain a healthy weight, reduces the risk of developing noncommunicable diseases such as type 2 diabetes and obesity [1,2,4], and may improve mental well-being and increase academic performance [5,6]. Despite the known benefits, global rates of PA engagement are low, with 37% failing to meet guidelines [7]. Women are significantly less likely to meet PA guidelines than men (32% vs 23%, respectively) [7]. The lowest level of PA is seen in adolescent girls aged 11 to 17 years, with 85% failing to meet guidelines, compared to 78% of adolescent boys [7].

Adolescence is the transitional period from childhood to adulthood, characterized by rapid growth and changing social expectations [8,9]. The period of adolescence is generally considered to be between the ages of 13 and 17 years; therefore, health interventions seeking to improve adolescent health often focus on this age group [3,8,9]. Conversely, the United Nations Children’s Fund (UNICEF) defines adolescence as individuals aged 10 to 19 years [10], with recent research suggesting that the period of adolescence should be extended further to include those aged 10 to 24 years [9]. Sawyer et al [9] suggest that a focus on “young people,” which would include traditional adolescents (aged 13-17 y) and young adults (aged 18-24 y), would account for varying growth patterns and changes in the timing of social role transitions across different countries. Another consideration is the development of lifelong PA habits. Young people move through 2 transitional periods: starting secondary school and then higher education [1,11,12]. It is during this period that individuals become responsible for their own health and develop their PA beliefs and behaviors, which generally remain consistent across the rest of their lifespan [1,11-13]. Although adolescents and young adults have different experiences, focusing on both age groups targets the transitional periods in which PA engagement decreases. For these reasons, this review is focused on young women aged 13 to 24 years.

Given the known benefits of PA and the importance of developing healthy habits during adolescence, there have been numerous interventions seeking to increase PA engagement during this life stage [14-18]. These interventions are primarily aimed at individuals aged <18 years, with none examining those aged 13 to 24 years specifically [14,16-18]. They also generally focus on both male and female individuals within the target age range [14,16-18]. One review [16] of school-based PA interventions identified a small increase in PA. Another review of 39 reviews of child and adolescent PA interventions reported a small positive effect [14]; however, the review noted that the positive impact of the interventions was small.

As previous PA interventions seeking to improve young women’s PA have had limited success, a different approach may be required [2,14,16,17,19-21]. One area of interest is the use of technology-supported PA as a strategy to increase PA [22-26]. Technology has been used as a tool for health promotion since the first mobile fitness apps were released in 2010, and technology-supported PA use increased during the COVID-19–related lockdowns [24,27-30]. Technology-supported PA can be defined as the use of some form of interactive technology or digitally accessed information to promote PA through (1) the demonstration of PA (eg, prerecorded or live-streamed fitness classes), (2) interaction with a device that provides feedback (eg, smartphones and wearable fitness trackers), and (3) interaction with fitness professionals (eg, web-based personal training) or other users of technology-supported PA (eg, through a fitness app or social media platform) [24,27,29,30]. Technology-supported PA is either self-led, with individuals using the technology in their own time (eg, apps and wearable fitness trackers), or facilitated, with sessions conducted by fitness professionals (eg, personal trainers or yoga instructors) in real time, allowing trainers to interact with their clients directly [24,27,29,30].

Recent systematic reviews focusing on the general population have suggested that technology-supported PA use is associated with increased PA and that the most successful types used behavior change techniques; were easy to use; and included gamification, such as offering some competition or challenge [8,18,22,25,31]. These reviews have generally focused only on a single form of technology (eg, apps or wearable fitness trackers). The level of effectiveness of technology-supported PA in increasing PA varies, with some studies reporting an overall significant improvement in PA for intervention groups compared to comparison groups [32,33] and others reporting no change in PA [34]. Reviews that did report improvements in PA, such as the studies by Champion et al [32] (22 publications involving 18,873 participants) and Lee et al [33] (16 interventions), noted that only a few of the included studies had postintervention follow-ups, and when they were included, it seemed that the improvements were not maintained [32,33].

In addition to the potential benefits for young women’s PA engagement, technology-supported PA could also improve young women’s physical literacy (PL) [35-37]. PL is a holistic approach to health that goes beyond simply engaging in PA; rather, it is focused on developing the skills, behaviors, and confidence needed to lead an active life [36,38,39]. PL is complex in nature, and it is defined and conceptualized in various ways across the globe [35-37,40]. One of the most comprehensive understandings of PL is provided in the Australian Physical Literacy Framework [38]. This framework groups the elements needed to improve PL into 4 domains: physical (eg, strength and movement skills), psychological (eg, confidence and motivation), social (eg, relationships and collaboration), and cognitive (eg, content knowledge and reasoning) [38]. Emerging research suggests that the development of PA habits is tied to the development of PL [18,35,38,39]. Studies seeking to improve PL have focused primarily on school-age children, with none examining young women specifically [35,41,42]. No review has examined whether technology-supported PA could impact each domain of PL [8,18,22,25].

### Objectives

Further research is needed to investigate the effectiveness of technology-supported PA use by young women and to identify the types of technology that may facilitate increased PA engagement and improve PL. The purpose of this systematic review was to investigate, in young women aged 13 to 24 years, the associations between different types of technology-supported PA and (1) PA engagement and (2) PL.

## Methods

### Overview

The selection of studies, analysis of data, and reporting of study results were conducted in accordance with the PRISMA (Preferred Reporting Items for Systematic Reviews and Meta-Analyses) guidelines [43]. The study was registered with PROSPERO in December 2022 (CRD42022382471), and there were no deviations from this protocol.

### Search Strategy

We conducted a systematic search of 6 databases: MEDLINE Complete, SPORTDiscus, Global Health, Education Source, Applied Science and Technology Source, and Embase. These databases were selected with advice from the Deakin University librarian due to their alignment with the review objectives. The search focused on articles published between January 1, 2010, and April 24, 2024. The strategy combined synonyms for “young women,” “technology-supported physical activity,” and “physical activity” (refer to Multimedia Appendix 1 for a full list of the terms used in the search), with truncation used to maximize search results.

### Eligibility Criteria

Eligible studies included randomized controlled trials (RCTs), nonrandomized interventions or retrospective observational studies investigating the effectiveness of technology-supported PA, as well as longitudinal and cross-sectional studies investigating the potential correlation between technology-supported PA use and PA engagement. Articles that were peer reviewed, contained original research, and published in English after 2010 were considered. The period from 2010 onward was selected because this year marked the release of the first PA mobile apps [44]. Studies that focused on women aged between 13 and 24 years were the primary target. The reference lists of the included articles were also searched for studies that may have been missed in the initial literature search.

### Study Selection and Screening

Studies were imported into Covidence (Veritas Health Innovation Ltd) [45] for screening. Qualitative studies, duplicates, articles not available in English, opinion articles, conference abstracts, systematic reviews, and study protocols were excluded. These study types were excluded because they either did not provide original data on the effectiveness of technology-supported PA or, in the case of conference abstracts and non-English articles, did not provide sufficient detail for data analysis. Full-text inclusion and exclusion criteria (Multimedia Appendix 2) were applied by the research team to identify articles for data extraction. Search terms related to PL were not included in the database search because the review focused on interventions that increased PA as the primary outcome of interest; in our analysis, we investigated whether these interventions included the elements of PL. Studies were excluded if they did not provide data on the target population, did not focus on increasing PA, or involved technology targeting specialized populations or those with chronic conditions. However, we included interventions focused on decreasing obesity because preliminary searches indicated that one of the main aims of obesity interventions was increasing PA, while interventions for other chronic conditions did not typically have PA as a key focus. In cases where the sample was the target age and included both male and female participants, studies were excluded if the results for young women were not reported separately.

### Data Extraction and Synthesis

Extraction was conducted using Covidence and Microsoft Excel. Extracted data included author, country, intervention setting, study design, theoretical framework, participant characteristics and demographic information, study aims, PA measurement methods, and the data collection tools used. For the intervention studies, information on the duration of the intervention and any follow-up conducted was also extracted. Details of the intervention and any control group were included, as was the method of data analysis. Finally, information was extracted on the effectiveness of the intervention and the effect size of any changes (if these were reported). Title and abstract screening and data collection were conducted by at least 2 authors independently. Disagreements on inclusion were discussed with a third author as needed. Before the full data extraction process, we extracted data from a sample of the studies and compared the findings to ensure consistency with data extraction.

To assess the effectiveness of the interventions and the reported associations between the use of technology-supported PA and PA in the cross-sectional studies, the reviewers divided the studies into 3 categories of technology-supported PA: interactive website or social media platforms, PA-tracking mobile apps, and wearable fitness trackers. Analyses were then conducted based on the effectiveness of each category of technology on PA (improvement or positive association, decline or negative association, or null effect). Analyses of the effect that the interventions had on the category of PA measured (accelerometry, PA intensity, guideline adherence, energy expenditure, step count, time spent walking, at-home exercise sessions, and increased exercise) were also conducted. The effectiveness of the interventions was assessed using a scale adapted from work conducted by Page et al [46]. If ≤30% of the studies reported a positive effect on PA or PL, the impact was coded as “no likely effect” (“0”). If 31% to 60% of the studies reported improvements, these were coded as “uncertain” (“?”) [46]. Finally, in instances where ≥61% of the studies reported changes in the expected direction (either PA improvements or decreases in sedentary behavior), the results were coded as “positive” (“+”).

The included studies were assessed according to their alignment to any of the 30 elements of the Australian Physical Literacy Framework [38]. PL is a relatively new concept, and definitions regarding what it is and is not vary depending on the framework used [47]. In addition, many studies explore outcomes that can be considered PL without defining it as PL [48]. We agreed that outcomes related to changes in social support and self-efficacy would not be included as PL outcomes. Although some aspects of social support could relate to elements within the social domain of the Australian Physical Literacy Framework, and self-efficacy could be related to the framework’s psychological domain, they are not included as part of this particular framework [38].

Alongside assessing the effectiveness of the interventions on the PA of young women aged 13 to 24 years, the research team divided the sample into 2 subgroups: adolescents aged <19 years and young adults aged ≥19 years. This was to account for the potentially significant physical, physiological, social, and environmental differences between these subgroups as well as the different PA guidelines recommended for each age group to be considered physically active.

### Quality Assessment

Two authors independently assessed each publication for quality and risk of bias using the JBI Critical Appraisal tools [43]. Three checklists were used to account for the different study designs used in the included articles: RCT, cross-sectional, and quasi-experimental. To ensure consistency between the quality assessments and the reliability of the quality analysis, we discussed the steps we would take to ensure consistency across all checklists and the different study designs included in the review; for example, we adapted questions in the cross-sectional and quasi-experimental checklists slightly to make them more consistent with those in the RCT checklist. Originally, only the RCT checklist required an exploration of both the validity and reliability of the tools used, while the other checklists only required an investigation of reliability; however, this requirement was added to the cross-sectional and quasi-experimental checklists. Furthermore, the validity and reliability of the data collection methods was considered in reference to the target population of young women. Within the RCT checklist, some questions explored the blinding of both participants and assessors. We decided that participant blinding to treatment assignment would be considered only if the study explicitly stated that blinding occurred or if each group received some form of intervention, the rationale being that blinding would be likely in this scenario because there would be no reason for the researchers to inform participants whether they were in the intervention or control group. When it came to assessor blinding, this was only considered if data collection was conducted in person and not via online self-report. Each study was assessed using the most suitable JBI checklist, due to variation in study designs the research team felt that the quality of retrospective observational studies was better assessed with the cross-sectional checklist rather than the quasi-experimental one. So, while retrospective observational studies would be considered nonrandomized in analysis they were grouped with the cross-sectional studies for quality analysis. If agreement on study quality could not be reached, a third author resolved the discrepancy by undertaking an independent assessment of the publication, and a final decision was made by consensus among the 3 authors.

## Results

### Description of Studies

**Figure 1:**
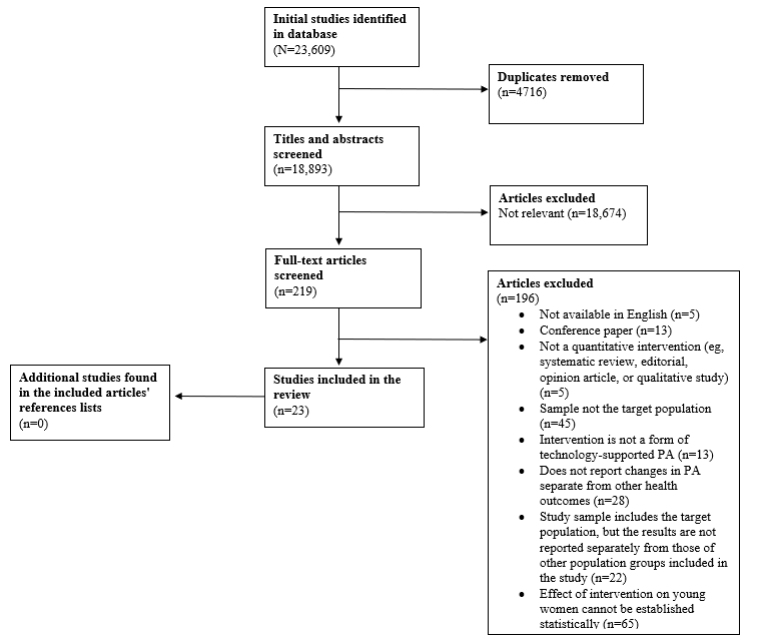
PRISMA (Preferred Reporting Items for Systematic Reviews and Meta-Analyses) flowchart.

The database searches yielded 23,609 records (Applied Science and Technology Source: n=1408, 5.96%; Education Source: n=6292, 26.65%; Embase: n=5432, 23.01%; Global Health: n=2336, 9.89%; MEDLINE Complete: n=5724, 24.24%; and SPORTDiscus: n=2417; 10.24%), of which 4716 (19.98%) were duplicates and removed, and 18,893 (80.02%) were screened by 2 authors based on title and abstract. At this stage, there were 23 disagreements that were resolved via discussion with the 2 authors who screened the papers. After title and abstract screening was completed, 219 articles (n=7, 3.2% involved disagreements that were resolved) were included in the full-text screening. Manual searches of the references lists of these articles were conducted, but no additional relevant articles were found (Figure 1).

### Study Characteristics

A total of 23 studies comprising 10,233 participants were included in the final review. Of these 23 studies, 18 (78%) involved interventions (RCTs: n=9, 50%; nonrandomized trials or retrospective observational studies: n=9, 50%): 12 (67%) included a control group (Multimedia Appendix 3) [40,49-59], and 6 (33%) involved a single sample (Multimedia Appendix 4) [60-65]. The remaining studies (5/23, 22%) were cross-sectional and investigated correlation between technology-supported PA and PA engagement (Multimedia Appendix 5) [66-70]. Of the 23 studies, 5 (22%) [40,60,62,63,69] explored variables that can be classified as elements of PL, although none mentioned PL specifically (Multimedia Appendix 6). Of these 5 studies, 4 (80%) included elements within the psychological domain [40,62,63,69] of the Australian Physical Literacy Framework [38], while 1 (20%) included elements within the framework’s physical domain [60].

Sample sizes ranged from 16 [60] to 4128 [68] participants. Of the 23 studies, 12 (52%) included only the target population [40,50-52,54,55,57,60-62,64,66], while the remaining 11 (48%) also included other populations, such as male participants, and individuals outside the target population age range [49,53,56,58,59,63,65,67-70]. Of the 23 studies, 9 (39%) included only adolescents [49,52,56-59,63,64,68], 12 (52%) focused on young adults [40,50,53-55,60-62,66,67,69,70], and 2 (9%) looked at both groups [51,65]; moreover, 6 (26%) sampled participants who were overweight or obese [40,52,54,55,61,62], and 3 (13%) focused on participants who were insufficiently active [51,58,64].

The types of technology-supported PA varied. Of the 18 interventions (RCTs: n=9, 50%; nonrandomized trials or retrospective observational studies: n=9, 50%), 4 (22%) used wearable fitness trackers [54,56,58,63], 10 (56%) used an interactive website or social media platform [40,49-51,53,59-62,64], and 4 (22%) used mobile apps [52,55,57,65].

Of the 4 wearable fitness tracker interventions, 3 (75%) were conducted with adolescents [56,58,63], and 1 (25%) was conducted with young adults [54].

Of the 10 interactive website or social media platform interventions, 3 (30%) targeted adolescents [49,59,64], 6 (60%) focused on young adults [40,50,53,60-62], and 1 (10%) focused on both groups [51].

Of the 4 interventions using mobile apps, 2 (50%) were conducted with adolescents [52,57], 1 (25%) was focused on young adults [55], and 1 (25%) focused on both groups [65].

Of the 18 intervention studies, 8 (44%) used forms of technology-supported PA that had been designed specifically for the study either as a stand-alone intervention or combined with a commercially available form of technology [49-53,58,61,62].

The cross-sectional studies compared various types of technology-supported PA, including mobile apps, and wearable fitness trackers [66-70]. Of the 5 cross-sectional studies, 1 (20%) focused on adolescents [68], and the other 4 (80%) involved young adults [66,67,69,70].

The most common intervention setting was universities [40,50,51,53-55,61,62,66,69,70], followed by high schools [49,52,56-59,63], while 5 (22%) of the 23 studies were conducted with the general population [60,64,65,67,68]. Of the 23 studies, 11 (48%) took place in the United States [51,53,54,59,61,62,64-67,69]; 2 (9%) were conducted in Australia [56,60]; and 1 (4%) study each was conducted in Saudi Arabia [40], the United Arab Emirates [50], Israel [49], China [70], Pakistan [55], Poland [52], Singapore [57], the Netherlands [58], and the United Kingdom [63]. Furthermore, 1 (4%) of the 23 studies compared data between Finland and Ireland [68].

Of the 23 studies, 9 (39%) reported being theory-based [50,53,54,58,61-64,66], although Kattelmann et al [53] and Melton et al [54] did not specify the theory on which their intervention was based. Social cognitive theory was the theory most commonly used (5/9, 56%) [50,61,62,64,66], with McFadden [66] combining it with the transtheoretical model of behavior change. The remaining studies (2/9, 22%) were based on self-determination theory [58,63]. Of the 9 theory-based studies, 3 (33%) focused on adolescents [58,63,64], and 6 (67%) involved young adults [50,53,54,61,62,66].

The most common method of data collection was self-report surveys, with 12 (52%) of the 23 studies using this method [40,49,52,53,58-60,66-70]. Of these 12 studies, 5 (42%) involved adolescents [49,52,58,59,68], and 7 (58%) focused on young adults [40,53,60,66,67,69,70]. Device-based data were collected in 5 (22%) of the 23 studies with an accelerometer [63,64], commercial wearable fitness tracker [54], or step-tracking commercial mobile app [55,65]. Both accelerometer studies were conducted with adolescents [63,64], tracking apps were used with young adults in 2 (40%) [54,55] of the 5 studies, and the remaining study (1/5, 20%) focused on both age groups [65]. Of the 23 studies, 6 (26%) used a combination of subjective and device-based measures [50,51,56,57,61,62]: 2 (33%) with adolescents [56,57] and 3 (50%) with young adults [50,61,62], while 1 (17%) investigated both groups [51].

Levels of PA were recorded as time spent in PA (days or minutes per week) in 8 (89%) of the 9 adolescent studies [49,52,56,58,59,63,64,68], 5 (42%) of the 12 studies with young adults [50,60,66,69,70], and 1 (50%) of the 2 studies that investigated all young women [51]. Both studies conducted on young adults by Joseph et al [61,62] combined days or minutes of weekly PA with interactive website engagement. Of the 4 studies that measured PA as total reported steps, 2 (50%) were conducted with young adults [54,55], 1 (25%) with adolescents [57], and 1 (25%) with both age groups [65]. The final tools of PA measurement—attendance at in-person PA sessions [40], total metabolic equivalents of task (METs) [53], and level of exercise engagement [67]—were all used in interventions focused on young adults.

A little more than half of the studies that included a control and comparison group (7/12, 58%) reported a positive effect on PA. Of these 7 studies, 4 (57%) were RCTs [51,52,54,58], and 3 (43%) were nonrandomized or retrospective observational studies (Multimedia Appendix 3) [40,49,50]. Of the 6 single-sample studies included in the review, 3 (50%) reported a positive outcome [61,64,65], 2 (33%) had no impact [60,62], and 1 (17%) reported a decrease in PA after the intervention (Multimedia Appendix 4) [63]. All cross-sectional studies included in the review (5/23, 22%) [66-70] reported a positive association between technology-supported PA use and PA engagement (Multimedia Appendix 5).

### Results of the Quality Assessment

The quality of the 23 studies varied, with 8 (35%) found to be of high quality [51,54,58,61,65,67,68,70], 7 (30%) rated as medium quality [49,52,53,56,59,60,62], and 8 (35%) determined to be low quality [40,50,55,57,63,64,66,69].

All RCTs included in the review (9/23, 39%; Multimedia Appendix 7) reported that true randomization was used to assign participants to the intervention and control groups, that the groups were treated identically, and that the outcome measurements used were the same for both groups [51-59]. However, some of the RCTs (4/9, 44%) did not provide information on participant or assessor blinding; of the 9 RCTs, only 2 (22%) reported that allocation to treatment groups was concealed [55,58], and only 3 (33%) used participant blinding [51,52,54]. No RCT reported whether those delivering the intervention were blind to the treatment group, although the question was not applicable in a little more than half of these RCTs (5/9, 56%) [51-55] because the intervention was self-led. In addition, no RCT stated whether the assessors measuring the outcomes of the intervention were blind to the treatment group. Of the 9 RCTs, 5 (56%) used self-report as the data collection method [51,53,54,57,58]; therefore, the question was not considered applicable. However, the remaining RCTs (4/9, 44%) did use outcome assessors and either reported that the assessors were not blinded [56] or did not report on it at all [52,55,59].

The quasi-experimental studies assessed with the relevant JBI checklist (Multimedia Appendix 7) were found to be of lower quality, but this was in part due to the variation in study designs. As such, questions related to the use of comparison or control groups only related to the studies by Al-Eisa et al [40], Ali et al [50], and Glaser et al [49]. Al-Eisa et al [40] and Ali et al [50] reported using a control group and assessing the intervention and control groups in the same way but did not provide information on the similarities between the 2 groups. Glaser et al [49] reported assessing the intervention and control groups in the same way but also provided information on the similarities between the groups at baseline, which was why the study was considered medium quality (56%).

All 8 quasi-experimental studies [40,49,50,60-64] outlined the independent and dependent variables under investigation, but none reported why the chosen method of data analysis was used. Only 3 (38%) of the 8 studies collected data at multiple points during the intervention period [60,61], and an intervention follow-up was only conducted by Joseph et al [61] and Larsen et al [64]. Only 1 (12%) of these 8 studies assessed with the JBI quasi-experimental checklist was found to be of high quality [61]. Of the overall 8 studies that were considered low quality, 4 (50%) were from this group [40,50,63,64].

Of the 6 cross-sectional and retrospective observational studies assessed with the relevant JBI checklist (Multimedia Appendix 7), 4 (67%) were considered high quality [65,67,68,70], while 2 (33%) were considered low quality [66,69]. All 6 studies provided details on the study participants and setting, the validity and reliability of the outcome measurements, and the appropriateness of the statistical analysis method used [65-70]. However, only half of the studies (3/6, 50%) clearly defined the participant inclusion criteria and reported whether objective standard criteria had been used for the measurement of the condition under investigation [65,68,70], and only 3 (50%) of the 6 studies identified potential confounding factors and explained how these factors had been addressed [67,68,70]. After conducting the quality assessment, we found that only Ng et al [68] and Wang et al [70] provided enough information to positively answer all checklist questions.

### Summary of the Findings

#### Associations With PA Outcomes

A summary of the study results according to the 3 types of technology-supported PA (interactive website or social media platform, PA-tracking mobile app, and wearable fitness tracker) and the type of PA measures assessed is provided in Table 1.

**Table 1:** Summary of physical activity (PA) results in the expected direction classified by type of PA measurement.

				Interactive website or social media platform									PA-tracking mobile app									Wearable fitness tracker										Proportion of positive findings
				Positive^a^		Negative^a^			Null^a^			Positive				Negative			Null		Positive				Negative			Null				
**Accelerometry**																																
	Accelerometry counts			—^b^		Larsen et al [64], 2018^c^			Joseph et al [62], 2016^c^			—				—			—		—				—			Melton et al [54], 2016^d^; Ridgers et al [56], 2021^d^			0/4	
	Total			0		1			1			0				0			0		0				0			2			0/4 (0%); positive outcomes code: 0^e^	
**PA intensity and duration**																																
	**Light**																															
		Min/wk	—		—			—			—				—			—		—				—			Slootmaker et al [58], 2010^d^			0/1		
	**Moderate**																															
		D/wk	Ali et al [50], 2021^d^		—			Whittemore et al [59], 2013^d^			—				—			—		—				—			—			1/2		
		Min/d	—		—			Ali et al [50], 2021^d^			—				—			—		—				—			Ridgers et al [56], 2021^d^			0/2		
		Min/wk	—		—			Curtis et al [60], 2020^c^; Kattelmann et al [53], 2014^d^; Papalia et al [69], 2018^f^			Wang et al [70], 2019^f^				—			Seah and Koh [57], 2021^d,g^		Slootmaker et al [58], 2010^d^				—			—			2/6		
	**Vigorous**																															
		D/wk	Ali et al [50], 2021^d^		—			Whittemore et al [59], 2013^d^			—				—			—		—				—			—			1/2		
		Min/d	Ali et al [50], 2021^d^		—			—			—				—			—		—				—			—			1/1		
		Min/wk	Kattelmann et al [53], 2014^d^; Papalia et al [69], 2018^f^		—			Curtis et al [60], 2020^c^			Wang et al [70], 2019^f^				—			Seah and Koh [57], 2021^g^		—				—			Slootmaker et al [58], 2010^d^			3/6		
	**Moderate to vigorous**																															
		D/wk	Glaser et al [49], 2024^d^		—			—			Dzielska et al [52], 2020^d,h^				—			—		—				—			—			2/2		
		Min/d	—		—			—			—				—			—		—				Kerner et al [63], 2019^c^			—			0/1		
		Min/wk	Larsen et al [64], 2018^c^		—			Joseph et al [61], 2015^c^; Joseph et al [62], 2016^c^			—				—			—		—				—			Slootmaker et al [58], 2010^d^			1/4		
	Total			7		0			9			3				0			2		1				1			4			11/27 (41%); positive outcomes code: ?^i^	
**PA guideline adherence**																																
	Meeting American College of Sports Medicine PA guidelines			—		—			—			—				—			—		McFadden [66], 2021^f^				—			—			1/1	
	Meeting World Health Organization PA guidelines			—		—			—			Ng et al [68], 2021^f^; Wang et al [70], 2019^f^				—			—		Ng et al [68], 2021^f^				—			—			3/3	
	Total			0		0			0			2				0			0		2				0			0			4/4 (100%); positive outcomes code: +^j^	
**Energy expenditure**																																
	Min/wk of total metabolic equivalents of task			—		—			Curtis et al [60], 2020^c^; Kattelmann et al [53], 2014^d^			—				—			Seah and Koh [57], 2021^d,g^		—				—			—			0/3	
	PA (heavy, kcal)			—		—			Cavallo et al [51], 2012^d^			—				—			—		—				—			—			0/1	
	PA (light, kcal)			—		—			Cavallo et al [51], 2012^d^			—				—			—		—				—			—			0/1	
	PA (moderate, kcal)			—		—			Cavallo et al [51], 2012^d^			—				—			—		—				—			—			0/1	
	PA (total, kcal)			—		—			Cavallo et al [51], 2012^d^			—				—			—		—				—			—			0/1	
	Total			0		0			6			0				0			1		0				0			0			0/7 (0%); positive outcomes code: 0	
**Step count**																																
	Total step count			—		—			—			Xian et al [65], 2017^c^				Memon et al [55], 2016^d^			Seah and Koh [57], 2021^d,g^		—				Melton et al [54], 2016^d^			—			1/4	
	Total			0		0			0			1				1			1		0				1			0			1/4 (25%); positive outcomes code: 0	
**Walking**																																
	D/wk			—		—			Ali et al [50], 2021^d^			—				—			Wang et al [70], 2019^f^		—				—			—			0/2	
	Min/d			Ali et al [50], 2021^e^		—			Curtis et al [60], 2020^c^			—				—			—		—				—			—			1/2	
	Min/wk			—		—			Kattelmann et al [53], 2014^d^			—				—			—		—				—			—			0/1	
	Total			1		0			3			0				0			1		0				0			0			1/5 (20%); positive outcomes code: 0	
**At-home exercise sessions**																																
	Number of at-home exercise sessions			Al-Eisa et al [40], 2016^d^		—			—			—				—			—		—				—			—			1/1	
	Total			1		0			0			0				0			0		0				0			0			1/1 (100%); positive outcomes code: +	
**Increased exercise**																																
	Increased exercise over the last year			—		—			—			Nagata et al [67], 2021^f^				—			—		—				—			—			1/1	
	Total			0		0			0			1				0			0		0				0			0			1/1 (100%); positive outcomes code: +	
Overall impact on PA				—		—	—			—				—			—						—			—			—	19/53 (36%); positive outcomes code: ?		

^a^Impact or association of study in the hypothesized direction.

^b^Not applicable.

^c^Intervention study with a single sample.

^d^Intervention study with comparison groups.

^e^0: no likely effect reported when ≤30% of the studies found changes in the expected direction (adapted from Page et al [46]).

^f^Cross-sectional study; shows correlation between technology and PA.

^g^Only looked at PA engagement on weekends.

^h^Only participants who were overweight or obese.

^i^Uncertain effect reported when 31% to 60% of the studies found changes in the expected direction (adapted from Page et al [46]).

^j^Positive effect reported when 61% to 100% of the studies found changes in the expected direction (adapted from Page et al [46]).

The included studies explored 53 different PA measures, which were grouped into 8 types: accelerometry, PA intensity and duration, PA guideline adherence, energy expenditure, step count, walking, at-home exercise sessions, and increased exercise. Most of the studies explored >1 type.

The most common PA outcome was self-reported intensity (15/23, 65%; 27 different analyses), which included both the type of PA, such as light, moderate, or vigorous, as well as the duration measured in minutes or days per week. Across these 15 studies [49,50,52,53,56-64,69,70], a positive effect or association was reported in 7 (47%; 11/27, 41% analyses); therefore, this was rated as uncertain in terms of effect (“?”).

Energy expenditure was the next most common outcome (4/23, 17%; 7 different analyses), which included METs and the number of calories expended per PA type. Of the 4 studies that included this measure, 3 (75%) measured minutes per week of METs [53,57,60], while Cavallo et al [51] measured PA in terms of kcal. The results of these interventions indicated that technology-supported PA had no likely effect on participant PA [51,53,57,60]; thus, this was rated as “0.”

Walking was measured as self-reported days or minutes per week or minutes per day of PA in 3 (17%) of the 18 intervention studies [50,53,60] and 1 (20%) of the 5 cross-sectional studies [70]. Of these 4 studies, only 1 (25%) reported that the use of technology-supported PA had a positive effect [50], while 3 (75%) reported a null result [53,60,70]; thus, this was rated as having no effect (“0”).

Adherence to PA guidelines (4 different analyses) was used as a measure in 3 (60%) of the 5 cross-sectional studies [66,68,70]. The guidelines used were those provided by the WHO [68,70] and the American College of Sports Medicine [66]. All forms of technology-supported PA measured were associated with greater adherence to PA guidelines [66,68,70]; therefore, this was rated as having a positive effect (“+”).

The overall positive impact of technology-supported PA was uncertain because only 36% (19/53) of the analyses reported a positive effect or changes in the expected direction.

#### Associations by Type of Technology-Supported PA

When comparing different types of technology-supported PA, data synthesis suggests that interactive websites or social media platforms (8/27, 30% analyses) and wearable fitness trackers (2/11, 18% analyses) had no likely effect on PA. The effect of mobile apps was more promising, but the full impact was uncertain (9/15, 60% analyses). A summary of these findings is reported in Table 2.

**Table 2:** Summary of physical activity (PA) results in the expected direction classified by type of intervention.

Type of intervention	Improvement or positive association reported in PA	Decline or negative association reported in PA	Null result in PA	Summary of results in the expected direction: analyses, n/N (%)	Code
Interactive website or social media platform	Intervention study with comparison groups: Al-Eisa et al [40], 2016^a^; Ali et al [50], 2021^b,c,d^; Kattelmann et al [53], 2014^c^; Glaser et al [49], 2014^e^ Intervention study with a single sample: Larsen et al [64], 2018^e^ Cross-sectional study: Papalia et al [69], 2018^c^	Intervention study with a single sample: Larsen et al [64], 2018^f^	Intervention study with comparison groups: Ali et al [50], 2021^b,d^; Cavallo et al [51], 2012^b,g,h^; Kattelmann et al [53], 2014^b,d,i^; Whittemore et al [59], 2013^b,c^ Intervention study with a single sample: Curtis et al [60], 2020^b,c,d,i^; Joseph et al [61], 2015^e^; Joseph et al [62], 2016^e,f^ Cross-sectional study: Papalia et al [69], 2018^b^	8/27 (30)	0^j^
PA-tracking mobile app	Intervention study with comparison groups: Dzielska et al [52], 2020^e,k^ Intervention study with a single sample: Xian et al [65], 2017^l^ Cross-sectional study: Ng et al [68], 2021^m,n^; Nagata et al [67], 2021^o^; Wang et al [70], 2019^b,c,m^	Intervention study with comparison groups: Memon et al [55], 2016^l^	Intervention study with comparison groups: Seah and Koh [57], 2021^b,c,i,l^ Cross-sectional study: Wang et al [70], 2019^d^	9/15 (60)	?^p^
Wearable fitness tracker	Intervention study with comparison groups: Slootmaker et al [58], 2010^b^ Cross-sectional study: McFadden [66], 2021^q^	Intervention study with comparison groups: Melton et al [54], 2016^l^ Intervention study with a single sample: Kerner et al [63], 2019^e^	Intervention study with comparison groups: Melton et al [54], 2016^f^; Ridgers et al [56], 2021^b,f^; Slootmaker et al [58], 2010^g,c,e^ Cross-sectional study: Ng et al [68], 2021^m^	2/11 (18)	0
Overall impact	—^r^	—	—	19/53 (36)	?

^a^Number of home exercise sessions.

^b^Moderate PA.

^c^Vigorous PA.

^d^Walking.

^e^Moderate to vigorous PA.

^f^Accelerometry counts.

^g^Light PA.

^h^Heavy PA.

^i^Metabolic equivalent of task.

^J^0: no likely effect reported when ≤30% of the studies found changes in the expected direction (adapted from Page et al [46]).

^k^Improvement seen only in participants who were overweight or obese.

^l^Total step counts.

^m^Meeting World Health Organization PA guidelines.

^n^Investigated multiple forms of technology-supported PA.

^o^Increased exercise in the previous year.

^p^Uncertain effect reported when 31% to 60% of the studies found changes in the expected direction (adapted from Page et al [46]).

^q^Meeting American College of Sports Medicine PA guidelines.

^r^Not applicable.

#### Associations With PL Outcomes

The elements of PL were explored in 4 (40%) of the 10 interactive website or social media platform studies [40,60,62,69] and in 1 (25%) of the 4 wearable fitness tracker studies (Multimedia Appendix 6) [63]. Improvements were reported in 2 (40%) [40,69] of these 5 studies for motivation [40] and engagement or enjoyment [69], while the other 3 (60%) [60,62,63] reported that the intervention had no effect. Overall, there was no effect on PL (2/7, 29% analyses; Multimedia Appendix 6).

#### Differences in the Effectiveness of Technology-Supported PA Between Adolescent and Young Adult Subgroups

Of the 15 studies that reported a positive impact or association between the use of technology-supported PA and PA engagement, 8 (52%) were conducted with young adults [40,50,54,61,66,67,69,70]. By comparison, only 33% (5/15) of the effective interventions focused on adolescents, while both the interventions that looked at both groups reported a positive result [51,65].

Mobile apps were only associated with positive PA outcomes when used by adolescents or across both age groups [65]. Both age groups reported positive PA outcomes when using interactive website or social media platforms (2/5, 40% with adolescents; 3/5, 60% with young adults) [40,49,50,61,64], while the adolescent [58] and young adult [54] subgroup each had 1 wearable fitness tracker intervention that reported a positive outcome. Overall, 56% (5/9) of the adolescent and 67% (8/12) of the young adult interventions reported a positive outcome.

#### Effectiveness of Technology-Supported PA According to Study Quality and Theoretical Framework

Of the 23 studies included in the review, 9 (39%) reported being underpinned by a theoretical design [50,53,54,58,61-64,66]. Of these 9 studies, 6 (67%) reported a positive outcome or association [50,54,58,61,64,66]. Among the 6 effective theory-based studies, social cognitive theory was the theory most commonly used, with 4 (67%) interventions drawing from this theory [50,61,62,64] and the cross-sectional study by McFadden [66] combining it with the transtheoretical model of behavior change. However, of the 4 effective theory-based studies, only 1 (25%) was considered high quality [61], with the other 3 (75%) considered poor quality [50,64,66]. The remaining effective theory-based studies (2/6, 33%) were considered high quality [54,58]; Slootmaker et al [58] used the self-determination theory, while Melton et al [54] did not provide information on the theory used.

Analysis of the non–theory-based interventions showed that 64% (9/14) had positive outcomes or associations between technology-supported PA use and PA engagement [40,49,51,52,65,67-70]. Of these 9 studies, 4 (44%) were cross-sectional studies [67-70]; therefore, only a positive association between technology-supported PA and PA use could be reported. Of the 9 effective non–theory-based interventions, 5 (56%) were considered high quality [51,65,67,68,70], 2 (22%) were considered medium quality [49,52], and 2 (22%) were rated poor quality [40,69] (Multimedia Appendix 7).

## Discussion

### Principal Findings

#### Overview

The primary aim of this review was to investigate the effectiveness of various types of technology-supported PA in increasing young women’s PA engagement. The secondary aim was to assess whether any of these interventions explored the elements of PL and whether the interventions led to improvements in PL. There were 3 main types of technology-supported PA investigated in these studies: mobile apps, wearable fitness trackers, and interactive websites or social media platforms. Analysis of the study findings did not indicate that technology-supported PA is an effective method of increasing young women’s PA, although, when breaking the findings down by age group, technology-supported PA may have a greater impact on PA engagement for young adults than for adolescents. There was no evidence that technology-supported PA is an effective way of increasing young women’s PL.

#### Effectiveness of Mobile Apps

While the overall impact of technology-supported PA on young women’s PA was uncertain, mobile apps may hold some promise, with positive results reported in 2 (50%) of the 4 studies [52,65] and 60% (9/15) of the measured PA outcomes. Currently, >80% of the global population own a mobile device, and these rates continue to increase [71]. Mobile phone use is especially prevalent among adolescents, with data indicating that in some countries, up to 95% of those aged 13 to 19 years have a mobile device, and individuals in this age group report higher levels of daily use than other age groups [72]. This higher rate of ownership and use may make mobile apps aiming to improve levels of PA engagement more effective in those aged <19 years, although only 4 (17%) of the 23 studies in this review focused on mobile apps, highlighting how little research has been conducted on this form of technology-supported PA [52,55,57,65]. Another consideration when investigating the effectiveness of mobile apps is the use of various forms of “gamification” [22,31-33]. Gamification, such as offering some competition or challenge, has been linked to improved intrinsic motivation and higher levels of app engagement [22,31-33]. Both mobile app studies included in this review that reported improvements in PA used gamification [52,65]. This result is in line with previously conducted studies, with several systematic reviews reporting that gamified apps led to higher levels of PA engagement than apps without elements of gamification [22,31-33]. However, just because mobile apps are popular does not necessarily mean that they are effective [22,34,73-75]. A common limitation of PA mobile apps is a lack of evidence and theory-based design, which has been reported in several systematic reviews [22,34,73-75]. This limitation can be seen in the interventions included in this review because both the mobile app interventions that did not improve PA used commercial fitness apps without providing evidence of reliability or a theoretical framework [55,57].

#### Effectiveness of Interactive Websites or Social Media platforms

Interventions based on interactive websites or social media platforms were not found to be an effective way of improving young women’s PA. Only 30% (8/27) of the measured PA outcomes assessed in interventions using an interactive website or social media platforms reported a positive effect or changes in the expected direction. Social media platforms are reported to be well liked by young women and considered an effective way of improving PA facilitators such as motivation or social support [23,76-79]. Nevertheless, these findings suggest that while social media platforms are popular, it does not necessarily mean that they are effective. This disconnect between engagement and effectiveness has been explored in previous research [76,78]. The study conducted by Duplaga [78] reported that only 33% of young adults following fitness influencers on social media platforms engaged in regular PA, while a survey by Camacho-Miñano et al [76] (37 participants aged 13-17 y) reported that Instagram fitness groups were associated with negative outcomes such as body dissatisfaction [40,60,76,78].

#### Effectiveness of Wearable Fitness Trackers

The least effective form of technology-supported PA was wearable fitness trackers, with only 18% (2/11) of the measured PA outcomes reporting a positive effect or changes in the expected direction. Only 2 (50%) of the 4 wearable fitness tracker interventions reported a positive result [54,58], and 1 (25%) decreased participant PA [63]; the authors theorized that this decrease may be due to wearable fitness trackers only increasing external motivation rather than autonomous motivation, which promotes long-term behavior change [80]. The limited effectiveness of wearable fitness trackers could be due to the design of the devices used in the interventions. Of the 4 wearable fitness tracker interventions, 3 (75%) used commercial devices [54,56,63], which are often not theory or evidenced based [74]. This may mean that the wearable fitness tracker used might not produce significant improvements in PA, even if the intervention is otherwise well designed. A lack of compliance with the devices and insufficient wear time could also have been a factor. Of the 4 studies, 3 (75%) reported issues with wearable fitness tracker compliance, and previous research has reported that this is a common issue with wearable fitness tracker interventions [81,82]. Another consideration is the age group in which the wearable fitness tracker interventions were conducted. Of the 4 interventions, 3 (75%) focused on adolescents [56,58,63], but a recent study by Shandhi et al [83] reported that young adults were the age group most likely to own and use a wearable fitness tracker; therefore, it is possible that the limited effectiveness of wearable fitness trackers reported in this review is due to the interventions being conducted primarily with adolescents [54,56,58,63,83].

### Variation Between Study Designs

The results may not only be attributed to the type of technology-supported PA. The different characteristics of the studies, including study designs, PA outcomes, and measurement tools should be considered; for example, theory-based interventions are reported to result in better outcomes and have more generalizable findings than those that are not [84,85]. This was evident in the review findings because of the 14 studies that were effective, only 6 (43%) had a theoretical framework [50,54,58,61,64,66]. In comparison, only 3 (38%) of the 8 studies that were not effective, reported being theory-based [53,62,63], although it is important to remember that a study being based on a theoretical framework does not automatically make it well designed. This can be seen in the findings of this review; it was noted that only 3 (33%) of the 9 effective theory-based interventions were high quality [54,58,61]. Our findings also noted that studies in the review were more likely to report a positive outcome or association between technology-supported PA use and PA engagement if they were high quality, regardless of the study design [51,54,58,61,65,67,68,70]. This is in line with recent reviews of health research, which note that studies that report higher quality are more likely to report positive outcomes [86,87].

Another consideration is the method of data collection. In PA research, data are primarily collected via self-report or device-based measures (eg, pedometers and accelerometers), and both methods have benefits and limitations [81,82]. Self-report methods are low cost and easy to administer but are prone to recall bias and overreporting of PA [81,82]. While device-based methods reduce the risk of bias, they are limited by cost, reduced generalizability, incorrect use, and the fact that the devices may not be suitable for all types of PA [81]. An example of this can be seen in the study by Larsen et al [64], who reported an increase in self-reported moderate to vigorous PA (from 24.7 min to 79.4 min) but a decrease in device-measured moderate to vigorous PA (from 21.4 to 10.4 min), attributed to changes in the type of PA the sample was engaging in after the intervention that was not effectively measured by accelerometers [64].

### Impact on PL

There was little evidence that technology-supported PA could improve young women’s PL, with only 29% (2/7) of the PL elements assessed reporting a positive effect or changes in the expected direction. Only 2 (40%) of the 5 included studies reported positive changes in PL [40,69]. The remaining interventions (3/5, 60%) reported no likely effect on PL [60,62,63]. This is not surprising, given that these 3 interventions also failed to increase PA engagement [60,62,63]. Only 1 (20%) of the 5 PL interventions focused on adolescents [63]. The primary domain explored in these interventions was the psychological domain. This focus on the psychological domain makes sense, given that these elements are known facilitators of PA for young women [31,88-90]. It should be noted that although these studies were found to explore aspects of PL, none of them mentioned the term “physical literacy” specifically [40,60,62,63,69]. However, this was expected because PL is a newer concept and the Australian Physical Literacy Framework was only published in 2019 [38]. Only 2 (40%) of the 5 PL studies were published after this date [60,63].

### Strengths and Limitations

To our knowledge, this review is the first synthesis of the impact that various types of technology-supported PA could have on the PA of young women (aged 13-24 y). This is a critical period for health promotion because this age range is when young women’s PA begins to decrease, and lifelong PA habits are formed [1,2]. This review also investigated and compared different types of technology-supported PA, while other reviews have focused on a single form of technology such as mobile health or mobile apps [22,33,34]. Focusing on a single form of technology ignores the fast-paced changes in technological advances, especially in a post–COVID-19 world. This is also the first review to investigate the potential impact that technology-supported PA could have on PL. The review was further strengthened by the use of a comprehensive quality assessment. It was also written in accordance with the PRISMA 2020 checklist (Multimedia Appendix 8). Alongside these strengths, there are also some limitations. The first is the inclusion of several single-sample studies because these do not have the same high quality found in RCTs. In addition, several cross-sectional studies were included; these cannot provide a causal link between technology-supported PA use and PA engagement. However, given the limited data available on the use of technology-supported PA by the target population, valuable insights would have been missed if the review had only included RCT studies. The variety of study designs included in this review also made assessing the quality of the studies more complex. Three different quality checklists were used in this review. The risk of this impacting the results was mitigated because we agreed on ways to make the different checklists more consistent. The analysis of the results of the included interventions could also have been hindered by the number of different PA measurements. There were 53 different ways in which PA outcomes were measured; therefore, we sought to address this by combining the different measurements into subgroups. The reported positive impact of mobile apps must be considered with caution due to the small number of studies (4/23, 17%) that focused on this form of technology-supported PA. It is possible that mobile apps may only seem to be more effective than interactive websites or social media platforms and wearable fitness trackers because the limited outcomes measured skewed the results. Another consideration is the poor quality of the included studies and a lack of theory-based design. We cannot be certain that the findings reported in this review are due to the limited effectiveness of technology-supported PA. It may instead be related to the quality of the studies and the types of technology-supported PA used. The findings of this review would have been strengthened if a meta-analysis of the findings could have been conducted; however, the variation between study designs, the measurement of PA outcomes, and study quality did not make this viable.

### Future Research

The findings of this review suggest 2 areas for future research. First, more research focused on young women’s use of technology-supported PA is needed. Only 12 (52%) of the 23 studies in this review examined young women specifically [40,50-52,54,55,57,60-62,64,66]. Previous research reports that young women experience unique barriers to PA that may not be targeted in interventions involving wider populations [60,89-91]. The second area that requires investigation is the potential of facilitated technology-supported PA. This review was unable to investigate the effectiveness of facilitated technology-supported PA because it was not used in any of the included studies. This finding was not surprising because facilitated technology-supported PA was not common before the COVID-19 pandemic. However, its use increased significantly during the pandemic, and it has continued to remain popular [27,30,92]. Emerging research indicates that facilitated technology-supported PA is most popular with young women, and fitness professionals see it as an effective method of client engagement [27,92]. Another consideration when addressing these areas is how researchers choose to measure PA. Greater consistency in the type of PA measured when investigating technology-supported PA would make comparison between interventions more effective.

### Conclusions

This is the first systematic review exploring the use of various types of technology-supported PA. It highlighted that there is no evidence yet for the benefits of technology-supported PA for young women. The review also highlighted how little research has been conducted in this area. Many of the studies included in this review were of poor quality and not grounded in theory; in addition, none investigated facilitated, technology-supported PA. Nevertheless, some of our findings indicate areas of promise for the future. Future interventions could focus on mobile apps because they may be more effective than interactive website or social media platforms and commercial wearable fitness trackers. Furthermore, interventions that combine multiple forms of support, such as mentors or in-person instruction, may be more effective than a single form of technology-supported PA. Adolescents and young adults may experience different barriers and facilitators to PA and the use of technology-supported PA. This must be considered when conducting research on this age group because it may be that different forms of technology are needed for each subgroup of young women. The review findings noted that interventions that were theory-based may be more effective than those that were not. Researchers should consider developing interventions underpinned by behavior change theory, with follow-ups after the intervention to see whether improvements in PA and the use of technology-supported PA have been maintained. In addition, more research is needed on the impact that technology-supported PA could have on adolescents because the review findings of the impact on this age group are very limited. This will not only improve the health outcomes of young women in the short term but also help them develop the skills and confidence needed to engage in PA across the lifespan. As technology-supported PA continues to improve and become more common, there is a greater need for well-designed evidence-based research exploring the impact that these new types of technology could have on young women’s PA and PL.

## Appendix

Multimedia Appendix 1 Search strategy.

Multimedia Appendix 2 Inclusion and exclusion criteria.

Multimedia Appendix 3 Intervention and result details from comparison group studies.

Multimedia Appendix 4 Intervention and result details from single-sample studies.

Multimedia Appendix 5 Result details from cross-sectional studies.

Multimedia Appendix 6 Summary of results in the expected direction by domain and element of physical literacy.

Multimedia Appendix 7 Quality assessment for each study design.

Multimedia Appendix 8 PRISMA (Preferred Reporting Items for Systematic Reviews and Meta-Analyses) checklist.

## Abbreviations

MET: metabolic equivalent of task
PA: physical activity
PL: physical literacy
PRISMA: Preferred Reporting Items for Systematic Reviews and Meta-Analyses
RCT: randomized controlled trial
UNICEF: United Nations Children’s Fund
WHO: World Health Organization

## References

[1] Feil K, Allion S, Weyland S, Jekauc D (2021). A systematic review examining the relationship between habit and physical activity behavior in longitudinal studies. Front Psychol.

[2] Posadzki P, Pieper D, Bajpai R, Makaruk H, Könsgen N, Neuhaus AL, Semwal M (2020). Exercise/physical activity and health outcomes: an overview of Cochrane systematic reviews. BMC Public Health.

[3] (2020). WHO guidelines on physical activity and sedentary behaviour (contract no.: CC BY-NC-SA 3.0 IGO). World Health Organization.

[4] The Lancet Public Health (2019). Time to tackle the physical activity gender gap. Lancet Public Health.

[5] Andermo S, Hallgren M, Nguyen TT, Jonsson S, Petersen S, Friberg M, Romqvist A, Stubbs B, Elinder LS (2020). School-related physical activity interventions and mental health among children: a systematic review and meta-analysis. Sports Med Open.

[6] Biddle SJ, Ciaccioni S, Thomas G, Vergeer I (2019). Physical activity and mental health in children and adolescents: an updated review of reviews and an analysis of causality. Psychol Sport Exerc.

[7] Global action plan on physical activity 2018-2030: more active people for a healthier world (contract no.: CC BY-NC-SA 3.0). World Health Organization.

[8] Rossi L, Behme N, Breuer C (2021). Physical activity of children and adolescents during the COVID-19 pandemic-a scoping review. Int J Environ Res Public Health.

[9] Sawyer SM, Azzopardi PS, Wickremarathne D, Patton GC (2018). The age of adolescence. Lancet Child Adolesc Health.

[10] (2022). Adolescent development and participation. UNICEF.

[11] Hawlader MD, Mozid NE, Sharmin S, Monju IH, Ahmed SB, Sarker W, Amin MA, Jhumur SS, Dalal K (2022). The art of forming habits: applying habit theory in changing physical activity behaviour. J Public Health (Berl.).

[12] Marginson S (2022). Research on international and global higher education: six different perspectives. Oxf Rev Educ.

[13] Park AH, Zhong S, Yang H, Jeong J, Lee C (2022). Impact of COVID-19 on physical activity: a rapid review. J Glob Health.

[14] Messing S, Rütten A, Abu-Omar K, Ungerer-Röhrich U, Goodwin L, Burlacu I, Gediga G (2019). How can physical activity be promoted among children and adolescents? A systematic review of reviews across settings. Front Public Health.

[15] Violant-Holz V, Gallego-Jiménez MG, González-González CS, Muñoz-Violant S, Rodríguez MJ, Sansano-Nadal O, Guerra-Balic M (2020). Psychological health and physical activity levels during the COVID-19 pandemic: a systematic review. Int J Environ Res Public Health.

[16] Owen MB, Curry WB, Kerner C, Newson L, Fairclough SJ (2017). The effectiveness of school-based physical activity interventions for adolescent girls: a systematic review and meta-analysis. Prev Med.

[17] Sims J, Scarborough P, Foster C (2015). The effectiveness of interventions on sustained childhood physical activity: a systematic review and meta-analysis of controlled studies. PLoS One.

[18] Yomoda K, Kurita S (2021). Influence of social distancing during the COVID-19 pandemic on physical activity in children: a scoping review of the literature. J Exerc Sci Fit.

[19] Amiri Farahani L, Asadi-Lari M, Mohammadi E, Parvizy S, Haghdoost AA, Taghizadeh Z (2015). Community-based physical activity interventions among women: a systematic review. BMJ Open.

[20] Cleland V, Granados A, Crawford D, Winzenberg T, Ball K (2013). Effectiveness of interventions to promote physical activity among socioeconomically disadvantaged women: a systematic review and meta-analysis. Obes Rev.

[21] Condello G, Puggina A, Aleksovska K, Buck C, Burns C, Cardon G, Carlin A, Simon C, Ciarapica D, Coppinger T, Cortis C, D'Haese S, De Craemer M, Di Blasio A, Hansen S, Iacoviello L, Issartel J, Izzicupo P, Jaeschke L, Kanning M, Kennedy A, Ling FC, Luzak A, Napolitano G, Nazare JA, Perchoux C, Pesce C, Pischon T, Polito A, Sannella A, Schulz H, Sohun R, Steinbrecher A, Schlicht W, Ricciardi W, MacDonncha C, Capranica L, Boccia S, DEDIPAC consortium (2017). Behavioral determinants of physical activity across the life course: a "DEterminants of DIet and Physical ACtivity" (DEDIPAC) umbrella systematic literature review. Int J Behav Nutr Phys Act.

[22] Angosto S, García-Fernández J, Valantine I, Grimaldi-Puyana M (2020). The intention to use fitness and physical activity apps: a systematic review. Sustainability.

[23] Bicen A, Uzunboylu H, Burgul NS (2020). Evaluation of participants’ opinions on online physical fitness training. J Sport Psychol.

[24] Füzéki E, Schröder J, Groneberg DA, Banzer W (2021). Online exercise classes during the COVID-19 related lockdown in Germany: use and attitudes. Sustainability.

[25] Mclaughlin M, Delaney T, Hall A, Byaruhanga J, Mackie P, Grady A, Reilly K, Campbell E, Sutherland R, Wiggers J, Wolfenden L (2021). Associations between digital health intervention engagement, physical activity, and sedentary behavior: systematic review and meta-analysis. J Med Internet Res.

[26] van Sluijs EM, Ekelund U, Crochemore-Silva I, Guthold R, Ha A, Lubans D, Oyeyemi AL, Ding D, Katzmarzyk PT (2021). Physical activity behaviours in adolescence: current evidence and opportunities for intervention. Lancet.

[27] Bratland-Sanda S, Mathisen TF, Sundgot-Borgen C, Sundgot-Borgen J, Tangen JO (2020). The impact of COVID-19 pandemic lockdown during spring 2020 on personal trainers' working and living conditions. Front Sports Act Living.

[28] Parker K, Uddin R, Ridgers ND, Brown H, Veitch J, Salmon J, Timperio A, Sahlqvist S, Cassar S, Toffoletti K, Maddison R, Arundell L (2021). The use of digital platforms for adults' and adolescents' physical activity during the COVID-19 pandemic (our life at home): survey study. J Med Internet Res.

[29] Dor-Haim H, Katzburg S, Revach P, Levine H, Barak S (2021). The impact of COVID-19 lockdown on physical activity and weight gain among active adult population in Israel: a cross-sectional study. BMC Public Health.

[30] (2020). COVID-19 - fitness industry impact report. Fitness Australia.

[31] Knight RL, McNarry MA, Sheeran L, Runacres AW, Thatcher R, Shelley J, Mackintosh KA (2021). Moving forward: understanding correlates of physical activity and sedentary behaviour during COVID-19-an integrative review and socioecological approach. Int J Environ Res Public Health.

[32] Champion KE, Parmenter B, McGowan C, Spring B, Wafford QE, Gardner LA, Thornton L, McBride N, Barrett EL, Teesson M, Newton NC, Health4Life team (2019). Effectiveness of school-based eHealth interventions to prevent multiple lifestyle risk behaviours among adolescents: a systematic review and meta-analysis. Lancet Digit Health.

[33] Lee AM, Chavez S, Bian J, Thompson LA, Gurka MJ, Williamson VG, Modave F (2019). Efficacy and effectiveness of mobile health technologies for facilitating physical activity in adolescents: scoping review. JMIR Mhealth Uhealth.

[34] Böhm B, Karwiese SD, Böhm H, Oberhoffer R (2019). Effects of mobile health including wearable activity trackers to increase physical activity outcomes among healthy children and adolescents: systematic review. JMIR Mhealth Uhealth.

[35] Liu Y, Chen S (2020). Physical literacy in children and adolescents: definitions, assessments, and interventions. Eur Phys Educ Rev.

[36] Longmuir PE, Tremblay MS (2016). Top 10 research questions related to physical literacy. Res Q Exerc Sport.

[37] Sum RK, Cheng CF, Wallhead T, Kuo CC, Wang FJ, Choi SM (2018). Perceived physical literacy instrument for adolescents: a further validation of PPLI. J Exerc Sci Fit.

[38] (2019). Australian physical literacy framework. Sport Australia.

[39] Sum RK, Ha AS, Cheng CF, Chung PK, Yiu KT, Kuo CC, Yu CK, Wang FJ (2016). Construction and validation of a perceived physical literacy instrument for physical education teachers. PLoS One.

[40] Al-Eisa E, Al-Rushud A, Alghadir A, Anwer S, Al-Harbi B, Al-Sughaier N, Al-Yoseef N, Al-Otaibi R, Al-Muhaysin HA (2016). Effect of motivation by "Instagram" on adherence to physical activity among female college students. Biomed Res Int.

[41] Carl J, Barratt J, Wanner P, Töpfer C, Cairney J, Pfeifer K (2022). The effectiveness of physical literacy interventions: a systematic review with meta-analysis. Sports Med.

[42] Filho VC, Pereira WM, Farias BD, Moreira TM, Guerra PH, Queiroz AC, Castro VH, Silva KS (2021). Scoping review on interventions for physical activity and physical literacy components in Brazilian school-aged children and adolescents. Int J Environ Res Public Health.

[43] Page MJ, Moher D, Bossuyt PM, Boutron I, Hoffmann TC, Mulrow CD, Shamseer L, Tetzlaff JM, Akl EA, Brennan SE, Chou R, Glanville J, Grimshaw JM, Hróbjartsson A, Lalu MM, Li T, Loder EW, Mayo-Wilson E, McDonald S, McGuinness LA, Stewart LA, Thomas J, Tricco AC, Welch VA, Whiting P, McKenzie JE (2021). PRISMA 2020 explanation and elaboration: updated guidance and exemplars for reporting systematic reviews. BMJ.

[44] Muntaner-Mas A, Martinez-Nicolas A, Lavie CJ, Blair SN, Ross R, Arena R, Ortega FB (2019). A systematic review of fitness apps and their potential clinical and sports utility for objective and remote assessment of cardiorespiratory fitness. Sports Med.

[45] Covidence systematic review software. Veritas Health Innovation.

[46] Page ZE, Barrington S, Edwards J, Barnett LM (2017). Do active video games benefit the motor skill development of non-typically developing children and adolescents: a systematic review. J Sci Med Sport.

[47] Edwards LC, Bryant AS, Keegan RJ, Morgan K, Cooper SM, Jones AM (2018). 'Measuring' physical literacy and related constructs: a systematic review of empirical findings. Sports Med.

[48] Essiet IA, Lander NJ, Salmon J, Duncan MJ, Eyre EL, Ma J, Barnett LM (2021). A systematic review of tools designed for teacher proxy-report of children's physical literacy or constituting elements. Int J Behav Nutr Phys Act.

[49] Glaser M, Green G, Barak S, Bord S, Levi S, Jakobovich R, Dunsky A, Zigdon A, Zwilling M, Tesler R (2024). The effects of the Friendship Online intervention program on physical activity, substance abuse, psychosomatic symptoms, and well-being among at-risk youth. J Adolesc.

[50] Ali HI, Attlee A, Alhebshi S, Elmi F, Al Dhaheri AS, Stojanovska L, El Mesmoudi N, Platat C (2021). Feasibility study of a newly developed technology-mediated lifestyle intervention for overweight and obese young adults. Nutrients.

[51] Cavallo DN, Tate DF, Ries AV, Brown JD, DeVellis RF, Ammerman AS (2012). A social media-based physical activity intervention: a randomized controlled trial. Am J Prev Med.

[52] Dzielska A, Mazur J, Nałęcz H, Oblacińska A, Fijałkowska A (2020). Importance of self-efficacy in eating behavior and physical activity change of overweight and non-overweight adolescent girls participating in healthy me: a lifestyle intervention with mobile technology. Nutrients.

[53] Kattelmann KK, Bredbenner CB, White AA, Greene GW, Hoerr SL, Kidd T, Colby S, Horacek TM, Phillips BW, Koenings MM, Brown ON, Olfert MD, Shelnutt KP, Morrell JS (2014). The effects of young adults eating and active for health (YEAH): a theory-based web-delivered intervention. J Nutr Educ Behav.

[54] Melton BF, Buman MP, Vogel RL, Harris BS, Bigham LE (2016). Wearable devices to improve physical activity and sleep. J Black Stud.

[55] Memon AR, Masood T, Awan W, Waqas A (2018). The effectiveness of an incentivized physical activity programme (active student) among female medical students in Pakistan: a randomized controlled trial. J Pak Med Assoc.

[56] Ridgers ND, Timperio A, Ball K, Lai SK, Brown H, Macfarlane S, Salmon J (2021). Effect of commercial wearables and digital behaviour change resources on the physical activity of adolescents attending schools in socio-economically disadvantaged areas: the RAW-PA cluster-randomised controlled trial. Int J Behav Nutr Phys Act.

[57] Seah ML, Koh KT (2020). The efficacy of using mobile applications in changing adolescent girls’ physical activity behaviour during weekends. Eur Phys Educ Rev.

[58] Slootmaker SM, Chinapaw MJ, Seidell JC, van MW, Schuit AJ (2010). Accelerometers and internet for physical activity promotion in youth’ feasibility and effectiveness of a minimal intervention [ISRCTN93896459]. Prev Med.

[59] Whittemore R, Jeon S, Grey M (2013). An internet obesity prevention program for adolescents. J Adolesc Health.

[60] Curtis RG, Ryan JC, Edney SM, Maher CA (2020). Can Instagram be used to deliver an evidence-based exercise program for young women? A process evaluation. BMC Public Health.

[61] Joseph RP, Dutton GR, Cherrington A, Fontaine K, Baskin M, Casazza K, Lorch D, Allison JJ, Durant NH (2015). Feasibility, acceptability, and characteristics associated with adherence and completion of a culturally relevant internet-enhanced physical activity pilot intervention for overweight and obese young adult African American women enrolled in college. BMC Res Notes.

[62] Joseph RP, Pekmezi D, Dutton GR, Cherrington AL, Kim Y, Allison JJ, Durant NH (2016). Results of a culturally adapted internet-enhanced physical activity pilot intervention for overweight and obese young adult African American women. J Transcult Nurs.

[63] Kerner C, Burrows A, McGrane B (2019). Health wearables in adolescents: implications for body satisfaction, motivation and physical activity. Int J Health Promot Educ.

[64] Larsen B, Benitez T, Cano M, Dunsiger SS, Marcus BH, Mendoza-Vasconez A, Sallis JF, Zive M (2018). Web-based physical activity intervention for Latina adolescents: feasibility, acceptability, and potential efficacy of the Niñas Saludables study. J Med Internet Res.

[65] Xian Y, Xu H, Xu H, Liang L, Hernandez AF, Wang TY, Peterson ED (2017). An initial evaluation of the impact of Pokémon GO on physical activity. J Am Heart Assoc.

[66] McFadden C (2021). Wearable exercise technology and the impact on college women’s physical activity. Quest.

[67] Nagata JM, Hazzard VM, Ganson KT, Hahn SL, Neumark-Sztainer D, Eisenberg ME (2022). Digital technology use and muscle-building behaviors in young adults. Int J Eat Disord.

[68] Ng K, Kokko S, Tammelin T, Kallio J, Belton S, O'Brien W, Murphy M, Powell C, Woods C (2020). Clusters of adolescent physical activity tracker patterns and their associations with physical activity behaviors in Finland and Ireland: cross-sectional study. J Med Internet Res.

[69] Papalia Z, Wilson O, Bopp M, Duffey M (2018). Technology-based physical activity self-monitoring among college students. Int J Exerc Sci.

[70] Wang T, Ren M, Shen Y, Zhu X, Zhang X, Gao M, Chen X, Zhao A, Shi Y, Chai W, Liu X, Sun X (2019). The association among social support, self-efficacy, use of mobile apps, and physical activity: structural equation models with mediating effects. JMIR Mhealth Uhealth.

[71] Olson JA, Sandra DA, Colucci ÉS, Al Bikaii A, Chmoulevitch D, Nahas J, Raz A, Veissière SP (2022). Smartphone addiction is increasing across the world: a meta-analysis of 24 countries. Comput Human Behav.

[72] Fortunato L, Lo Coco G, Teti A, Bonfanti RC, Salerno L (2023). Time spent on mobile apps matters: a latent class analysis of patterns of smartphone use among adolescents. Int J Environ Res Public Health.

[73] Carroll JK, Moorhead A, Bond R, LeBlanc WG, Petrella RJ, Fiscella K (2017). Who uses mobile phone health apps and does use matter? A secondary data analytics approach. J Med Internet Res.

[74] Paganini S, Terhorst Y, Sander LB, Catic S, Balci S, Küchler A, Schultchen D, Plaumann K, Sturmbauer S, Krämer LV, Lin J, Wurst R, Pryss R, Baumeister H, Messner E (2021). Quality of physical activity apps: systematic search in app stores and content analysis. JMIR Mhealth Uhealth.

[75] Panicker RM, Chandrasekaran B (2022). "Wearables on vogue": a scoping review on wearables on physical activity and sedentary behavior during COVID-19 pandemic. Sport Sci Health.

[76] Camacho-Miñano MJ, MacIsaac S, Rich E (2019). Postfeminist biopedagogies of Instagram: young women learning about bodies, health and fitness. Sport Educ Soc.

[77] Drehlich M, Naraine M, Rowe K, Lai SK, Salmon J, Brown H, Koorts H, Macfarlane S, Ridgers ND (2020). Using the technology acceptance model to explore adolescents' perspectives on combining technologies for physical activity promotion within an intervention: usability study. J Med Internet Res.

[78] Duplaga M (2020). The use of fitness influencers' websites by young adult women: a cross-sectional study. Int J Environ Res Public Health.

[79] Parker K, Gould L, Nand M, Rawstorn JC, Contardo Ayala AM, Maddison R, Toffoletti K (2022). Understanding Australian adolescent girls' use of digital technologies for healthy lifestyle purposes: a mixed-methods study. BMC Public Health.

[80] Ntoumanis N, Ng JY, Prestwich A, Quested E, Hancox JE, Thøgersen-Ntoumani C, Deci EL, Ryan RM, Lonsdale C, Williams GC (2021). A meta-analysis of self-determination theory-informed intervention studies in the health domain: effects on motivation, health behavior, physical, and psychological health. Health Psychol Rev.

[81] Pedišić Ž, Bauman A (2015). Accelerometer-based measures in physical activity surveillance: current practices and issues. Br J Sports Med.

[82] Steene-Johannessen J, Anderssen SA, van der Ploeg HP, Hendriksen IJ, Donnelly AE, Brage S, Ekelund U (2016). Are self-report measures able to define individuals as physically active or inactive?. Med Sci Sports Exerc.

[83] Shandhi MM, Singh K, Janson N, Ashar P, Singh G, Lu B, Hillygus DS, Maddocks JM, Dunn JP (2024). Assessment of ownership of smart devices and the acceptability of digital health data sharing. NPJ Digit Med.

[84] Atkins L, Francis J, Islam R, O'Connor D, Patey A, Ivers N, Foy R, Duncan EM, Colquhoun H, Grimshaw JM, Lawton R, Michie S (2017). A guide to using the theoretical domains framework of behaviour change to investigate implementation problems. Implement Sci.

[85] Heath G, Cooke R, Cameron E (2015). A theory-based approach for developing interventions to change patient behaviours: a medication adherence example from paediatric secondary care. Healthcare (Basel).

[86] Dal Santo T, Rice DB, Amiri LS, Tasleem A, Li K, Boruff JT, Geoffroy M, Benedetti A, Thombs BD (2023). Methods and results of studies on reporting guideline adherence are poorly reported: a meta-research study. J Clin Epidemiol.

[87] Pirosca S, Shiely F, Clarke M, Treweek S (2022). Tolerating bad health research: the continuing scandal. Trials.

[88] Dechrai IM, Mazzoli E, Hanna L, Morgan PJ, Young MD, Grounds JA, Kennedy S, Pollock ER, Barnett LM (2022). Are gender-stereotyped attitudes and beliefs in fathers and daughters associated with girls’ perceived motor competence?. Phys Educ Sport Pedagogy.

[89] Rosselli M, Ermini E, Tosi B, Boddi M, Stefani L, Toncelli L, Modesti PA (2020). Gender differences in barriers to physical activity among adolescents. Nutr Metab Cardiovasc Dis.

[90] Watson A, Eliott J, Mehta K (2015). Perceived barriers and facilitators to participation in physical activity during the school lunch break for girls aged 12–13 years. Eur Phys Educ Rev.

[91] Depper A, Howe PD (2016). Are we fit yet? English adolescent girls’ experiences of health and fitness apps. Health Sociol Rev.

[92] García-Fernández J, Gálvez-Ruiz P, Bohórquez MR, Grimaldi-Puyana M, Cepeda-Carrión I (2020). The relationship between technological capabilities and organizational impact: direct and indirect routes for employed and self-employed personal fitness trainers. Sustainability.

