# Supplementary material for: Technology-Supported Physical Activity and Its Potential as a Tool to Promote Young Women’s Physical Activity and Physical Literacy: Systematic Review
Source: J Med Internet Res. 2024 Oct 18;26:e52302. doi: 10.2196/52302 (PMC11530733; doi:10.2196/52302)
Supplement: Multimedia Appendix 1 [file jmir_v26i1e52302_app1.pdf]

Multimedia Appendix 1: Technology-supported physical activity and its potential as a tool to promote young women's physical activity and physical literacy: Systematic review search strategy.

**Concept 1**	Online OR Website OR App OR application OR technology OR virtual OR digital OR Wii OR Xbox OR PlayStation OR Nintendo OR Fitbit OR Smartwatch OR Smartphone OR Garmin OR cellphone OR phone OR email OR "Apple Watch" OR mobile OR "Fitness Pal" OR "Lets fit" OR Coros OR Oura OR Whoop OR Mob Voi OR Suunto OR Ignite OR Amazfit OR Samsung OR Withings
**Concept 2**	exercise OR fitness OR "physical activity" OR Yoga OR Pilates OR Boxing OR Cycling Running OR walking OR jogging cardio* weight* OR muscle OR resistance OR "personal training" OR gym OR Exercise OR "Exercise Movement Techniques"
**Concept 3**	Engage* OR usage OR use OR Awareness OR participat*
**Concept 4**	Girl* OR young OR adolesc* OR teen* OR female* OR wom?n OR "young women" OR student OR "high school*" OR college OR university

**Databases**	Applied Science and Technology Source, Education Source, Embase, MEDLINE Complete, Global Health, and SPORTDiscus
