# Supplementary material for: Technology-Supported Physical Activity and Its Potential as a Tool to Promote Young Women’s Physical Activity and Physical Literacy: Systematic Review
Source: J Med Internet Res. 2024 Oct 18;26:e52302. doi: 10.2196/52302 (PMC11530733; doi:10.2196/52302)
Supplement: Multimedia Appendix 2 [file jmir_v26i1e52302_app2.pdf]

Multimedia Appendix 2: Inclusion - Exclusion criteria: Technology-supported physical activity and its potential as a tool to promote young women's (13-24 years-old) physical activity and physical literacy: Systematic review.

Inclusion - criteria	Exclusion criteria
• Peer-reviewed<br>• Available in English<br>• Published after 2010<br>• Includes participants who are young women aged 13-24 (can be 2 years above or below this group)<br>• If only mean age is reported, then it must fall between 13-24 years-old.<br>• Interventions focused on weight reduction and the treatment of obesity<br>• Quantitative or mixed-method research that reports on the use of a form of technology-supported PA use<br>• Technology-supported PA uses some form of interactive technology or digitally accessed information to promote PA engagement either through either demonstration of PA, interaction or the potential to interact with fitness professionals or other users (e.g., social media)<br>• Includes interactive webpages (e.g., input PA data to compare to other participants), apps, smartphones, social media, online support groups, videos, online personal training, pre-recorded or live Zoom classes<br>• mHealth (a common term for technology-supported PA, usually mobile phone based)<br>• Fitness trackers such as Fitbit, Amazfit, Garmin, Xiaomi or Apple Watch<br>• Methods are either self-lead which people use in their own time or facilitated in which fitness professionals run it in real-time	• Not available in English<br>• Published before 2010<br>• Not the target population<br>• Qual studies<br>• Not an original study (e.g., systematic review)<br>• Not a form of technology-supported PA<br>• Data from wider studies not separated from other population groups included in the study<br>• Interventions focused on supporting special populations - disability, pregnancy, chronic conditions, specific illnesses<br>• Data was collected online but was not about technology-supported PA use specifically<br>• Research uses the wrong types of technology-supported<br>- Information read on webpages<br>- Digital newsletters<br>- Data is collected online but is not about technology-supported PA use specifically<br>- Qual data on acceptability<br>• These forms do not involve demonstration or interaction
