# Supplementary material for: Technology-Supported Physical Activity and Its Potential as a Tool to Promote Young Women’s Physical Activity and Physical Literacy: Systematic Review
Source: J Med Internet Res. 2024 Oct 18;26:e52302. doi: 10.2196/52302 (PMC11530733; doi:10.2196/52302)
Supplement: Multimedia Appendix 3 [file jmir_v26i1e52302_app3.pdf]

Multimedia Appendix 3. Intervention and result details for comparison group studies

**Author year, country**	**Sample**	**Other populations**	**Intervention**	**Control**	**Length**	**PA outcome**	**Data collection tool**	**Results**	**Effect size**
**Adolescents (<19 years)**									
**Dzielska et al [1] 2020, Poland**	N=1111<br>Age: 15 years – HI	No	Healthy Me intervention (Full) - Gamified mobile app and a fitness band for PA tracking. Messages and articles about health, and school-based in person workshops	a. Healthy Me intervention (Partial) – traditional mobile app and a fitness band for PA tracking. Messages and articles about health.<br><br>b. No intervention – traditional mobile app and fitness band for PA tracking	6 months, 3-month follow-up	Days per week of MVPA	PA question about days of MVPA as part of the Health behaviour index questionnaire developed for the study	**Days of MVPA (overweight)**<br>**Full intervention**<br>No days - 6.6% 4.9% 6.4%<br>1-3 days - 36.0% 38.7% 41.4%<br>4-6 days - 44.1% 41.5% 45.0%<br>7 days - 13.2% 14.8% 7.1%<br>**Part intervention**<br>No days - 5.1% 3.4% 10.2%<br>1-3 days - 54.2% 46.6% 44.1%<br>4-6 days - 35.6% 39.7% 35.6%<br>7 days - 5.1% 10.3% 10.2%<br>**Control**<br>No days - 1.7% 0.0% 3.4%<br>1-3 days - 46.6% 47.4% 51.7%<br>4-6 days - 46.6% 47.4% 41.4%<br>7 days - 5.2% 5.3% 3.4%<br><br>**Days of MVPA (normal weight)**<br>**Full intervention**<br>No days - 4.2% 4.0% 6.7%<br>1-3 days - 31.1% 39.5% 42.3%<br>4-6 days - 49.1% 41.2% 41.4%<br>7 days - 15.6% 15.3% 9.6%<br>**Part intervention**<br>No days - 5.8% 8.4% 8.5%<br>1-3 days - 39.7% 41.1% 51.3%<br>4-6 days - 43.9% 41.1% 33.3%<br>7 days - 10.6% 9.5% 6.9%<br>**Control**<br>No days - 2.0% 3.0% 4.5%<br>1-3 days - 36.1% 35.3% 36.6%<br>4-6 days - 47.0% 48.3% 52.0%<br>7 days - 14.9% 13.4% 6.9%	Not reported
**Glaser et al [2] 2023, Israel**	N=51<br>Age: 15-17 years HI	Boys N=129 Age: 15-17 years HI	Friendship Online Intervention Program (FOIP)<br>30-minute weekly Zoom calls with a mentor who provided support and education	No intervention	8 months, no follow-up	Days per week of 60+ minutes MVPA	Self-reported PA via online quiz	**Days of MVPA**<br>IV group: >4 days of MVPA increased 63.15% (χ^2 = 17.01, p <0.001)<br>C group: No change	Not reported
**Ridgers et al [3] 2021, Australia**	N= 151<br>Age: 13.7 years (SD 0.4) - HI	Boys N=137<br>Age: 13.7 years (SD 0.4) - HI	Fitbit Flex with mobile app for PA tracking and providing weekly PA challenges.<br><br>Access to a private Facebook group providing PA and health information	Waitlist control	12 weeks, 6-month follow-up	Days and minutes of MVPA<br><br>8-day Accelerometry Counts	Self-reported via a survey conducted by a RA<br><br>GT3X+ ActiGraph activity monitor	Accelerometry<br>**Immediately post-intervention MVPA**<br>0.4, 95% CI −4.66 to 5.56, P=0.86<br>**6-month MVPA difference**<br>-0.03, 95% CI −5.06 to 5.01, P=0.99<br><br>Self-report<br>**Immediately post-intervention MVPA difference**<br>0.4, 95% −0.12 to 0.85, P=0.14<br>**6-month MVPA difference**<br>0.3, 95% CI −0.21 to 0.77, P=0.27	Not reported

**Seah and Koh [4] 2021, Singapore**	N= 36<br>Age: 14.9 years (SD 0.30) - HI	No	Access to the MapMyFitness mobile app to promote and track PA on weekends	No intervention	4 weeks, no follow-up	Total steps<br><br>Minutes per week of MET, MPA and VPA	Step tracking mobile app.<br><br>3DPAR survey	**MET**<br>There were no significant differences reported<br><br>Data collected in week two that did report a significant difference t (34) = .276, P=0.784, based on the reviewer's assessment the P value provided is a likely mistype and the difference at week 2 is significant	Not reported
**Slootmaker et al [5] 2010, The Netherlands**	N= 55<br>Age: 15.1 years - IA	Boys<br>N= 32<br>Age: 15.1 years - IA	PAM COACH – Fitness tracker and interactive website with PA tracking and goal setting tools and individualised PA advice	A single brochure with general PA recommendations	3 months, 8-month follow-up	Days per week and minutes of MVPA	AQuAA survey	**MPA**<br>Increase of 411 minutes in IG (p<0.05 between groups); not sustained at follow-up<br><br>All other PA outcomes were considered non-significant.	Not reported
**Whittemore et al [6] 2013, USA**	N= 238<br>Age: 15.31 years (SD 0.69) - HI	Boys<br>N= 146<br>Age: 15.31 years (SD 0.69) - HI	HEALTH[e]TEEN + CST interactive website – PA tracking, goal setting, health coaching and interactive health lessons.<br><br>Coping skills training	HEALTH[e]TEEN interactive website – PA tracking, goal setting, health coaching and interactive health lessons	6 months no follow-up	Days per week of VPA, MPA and muscle strengthening	Exercise survey items of the Youth Risk Behaviours Survey	**VPA, MPA or muscle strengthening**<br>Non-significant differences between groups	Not reported
**Young adults (≥19 years)**									
**Al-Eisa et al [7] 2016, Saudi Arabia**	N=58<br>Age: 20.30 years (SD 0.96) - HI	No	Link to an at-home exercise video<br><br>Access to a private Instagram group where participants could share progress and motivate each other	Link to an at-home exercise video	4 weeks, no follow-up	Number of PA sessions completed at home	A tracking sheet listing all sessions completed	**Sessions of PA**<br>Performing >8 sessions ['adherent']:<br>IV group 17% vs. C group 4%<br>Mean intervention sessions:<br>IV group 3.83 ± 4.05 vs C group 2.09 ±2.29	Not reported
**Ali at al [8] 2021, United Arab Emirates**	N=246<br>Age: 21.94 years (SD 2.03) – OO	No	Rashakaty – Enhanced. Interactive website including information on PA, PA tracking tools, feedback, counselling, and participant challenges	Rashakaty – Basic.<br>A static website that contained educational material	16 weeks, no follow-up	Days and minutes of MPA, VPA MVPA and walking<br><br>Total Steps	IPAQ—Short Form<br><br>PACER app (steps)	**Days of VPA (IV group)**<br>Change score: Median 1 (-1-1) No P value provided.<br>**Minutes of VPA (IV group)**<br>Change score: Median 5 (0–26.25) (P = 0.025)<br>**Days of MPA (IV group)**<br>Change score: Median 1 (0–2) (P <0.001)<br>**Minutes of MPA (IV group)**<br>Change score: Median 10 (0–28.75) (P <0.001)<br>**Days of walking (IV group)**<br>Change score: Median 0 (-1–1) No P value provided.<br>**Minutes of walking (IV group)**<br>Change score: Median 15 (0–35) (P <0.001)<br>**Days of VPA (C group)**<br>Change score: Median 0 (-2-0) No P value provided.<br>**Minutes of VPA (C group)**<br>Change score: Median 0 (0-5) (P = 0.002)<br>**Days of MPA (C group)**	Not reported

								Change score: Median 0 (-1-0.75) No P value provided.<br>**Minutes of MPA(C group)**<br>Change score: Median 0 (0–22.5) No P value provided.<br>**Days of walking (C group)**<br>Change score: Median 1 (0–2.75) No P value provided.<br>**Minutes of walking (C group)**<br>Change score: Median -5 (-20–8) No P value provided.	
**Cavallo et al [9] 2012, USA**	N= 134 Age: <25 years – IA	No	Internet Support for Healthy Associations Promoting Exercise (INSHAPE). Interactive website including information on PA, PA tracking and goal setting tools.<br><br>Access to a private Facebook group for additional support and PA information	Internet Support for Healthy Associations Promoting Exercise (INSHAPE). Static website without PA tracking and goal setting tools.<br><br>PA information sent via email.	12 weeks no follow-up	Total kcal expended, kcal expended via PA that was deemed light, moderate, and heavy	Paffenbarger activity questionnaire adapted for online use	**PA (total kcal)**<br>2394.75 (SD 1448.00) vs. 2248.98 (SD 1541.19)<br>Group X time interaction effect = F (1, 127.75) = 0.42, P=0.52<br>Time effect = F (1, 127.75) =23.59, p=0.000<br>**PA (heavy kcal)**<br>151.79 (SD 333.57) vs. 298.21 (SD 575.32)<br>Group X time interaction effect = F (1, 129.58) = 0.35, P=0.55<br>Time effect F (1, 129.58) = 9.19, P=0.003<br>**PA (moderate kcal)**<br>253.79 (SD 646.08) vs. 378.23 (SD 731.36)<br>Group X time interaction effect = F (1, 128.75) =0.26, P=0.61<br>Time effect = F (1, 128.75) =6.80, p=0.01<br>**PA (light kcal)**<br>81.25 (SD 182.55) vs. 61.69 (SD 134.95)<br>Group X time interaction effect = F (1, 128.84) =0.69, P=0.41<br>Time effect = F (1, 128.84) =2.54, P=0.11	Not reported
**Kattelmann et al [10] 2014, USA**	N= 1098 Age: 19.3 years (SD 1.1) - HI	Men N= 541 Age: 19.3 years (SD 1.1) - HI	Project Yeah – interactive website with PA tracking, goal setting, email reminders and short health education lessons	No intervention	10 weeks, 15-month follow-up	Days and minutes of MET, VPA, MPA and walking	IPAQ	There were no reported differences between the IV and C groups for mean total MET, walking minutes per week of PA per week.<br><br>**MET**<br>Change in moderate MET minutes per week in both IV and C groups but no difference between them.<br><br>Females in the IV group had a significantly higher albeit small effect size (Cohen d = 0.2) in VPA<br>MET-minutes female C group. P=0.05<br><br>When comparing females only total mean MET minutes per week the IV group appeared to report higher levels than the C (1,132.9 SD 1,123 vs 880.0 SD 1227) but this was not sustained at follow-up (949.2 SD 1,092 vs. 897.2 SD 1087) with no P value provided	Cohen d effect size of 0.2 for women's weekly VPA compared to control
**Melton et al [11] 2016, USA**	N= 69 19.9 years (SD 1.7) – OO and AA	No	Jawbone UP fitness band and mobile app.<br><br>Weekly email reminders were sent with general health tips	MyFitnessPal mobile app.<br><br>Weekly email reminders were sent with general health tips	6 weeks, 8-week follow-up	Total steps<br><br>7-day Accelerometry Counts	Jawbone UP app and MyFitnessPal app<br><br>GT3X+ ActiGraph activity monitor	**Total steps**<br>**Post intervention (6 weeks)**<br>App: IV group =10,674 vs. C group=10,870) P=0.7<br>Accelerometry: IV group = 2,319 vs. C=2,415 P=0.42<br><br>**Follow-up (8 weeks)**<br>App: IV group=10,674 vs. C group=9,605) P=0.04<br>Accelerometry: IV group= 2,319 vs. C=2,251) P=0.64	Not reported

**Memon et al [12] 2016, Pakistan**	N= 56 Age: 20.63 years (SD 1.67) – OO	No	ActiveStudent trial: The Moves step tracking app combined with a financial incentive for engaging in PA	The Moves step tracking app without a financial incentive	5 weeks no follow-up	Total steps	MOVES mobile app	**Total steps**<br>IV group =57799.61 ±48416.33 vs. C group= 47314.36 ±29280.44 P=0.331	Not reported

Notes: 3DPAR- three-day physical activity recall, AA- African American, AQuAA- Activity Questionnaire for Adolescents and Adults, C - Control group, HI-Healthy individuals (not targeting any specific health concerns), IA- Insufficiently active, IPAQ- International Physical Activity Questionnaire, IV group- Intervention group, kcal- kilocalories, MET- Metabolic equivalent of task, MPA- Moderate physical activity, MVPA- Moderate to vigorous physical activity, OO-Overweight or Obese, PA- Physical activity, RCT-Randomised control trial, RA- Research assistant SD – Standard deviation, VPA- Vigorous physical activity

References

1. Dzielska A, Mazur J, Nalecz H, Oblacinska A, Fijalkowska A. Importance of Self-Efficacy in Eating Behavior and Physical Activity Change of Overweight and Non-Overweight Adolescent Girls Participating in Healthy Me: A Lifestyle Intervention with Mobile Technology. Nutrients. 2020 Jul 17;12(7). PMID: 32709005. doi: 10.3390/nu12072128.
2. Glaser M, Green G, Barak S, Bord S, Levi S, Jakobovich R, et al. The effects of the Friendship Online Intervention Program on physical activity, substance abuse, psychosomatic symptoms, and well-being among at-risk youth. J Adolesc. 2024 Feb;96(2):251-65. PMID: 37985148. doi: 10.1002/jad.12272.
3. Ridgers ND, Timperio A, Ball K, Lai SK, Brown H, Macfarlane S, et al. Effect of commercial wearables and digital behaviour change resources on the physical activity of adolescents attending schools in socio-economically disadvantaged areas: the RAW-PA cluster-randomised controlled trial. Int J Behav Nutr Phys Act. 2021 Apr 12;18(1):52. PMID: 33845853. doi: 10.1186/s12966-021-01110-1.
4. Seah MLC, Koh KT. The efficacy of using mobile applications in changing adolescent girls’ physical activity behaviour during weekends. European Physical Education Review. 2020;27(1):113-31. doi: 10.1177/1356336x20930741.
5. Slootmaker SM, Chinapaw MJ, Seidell JC, van Mechelen W, Schuit AJ. Accelerometers and Internet for physical activity promotion in youth? Feasibility and effectiveness of a minimal intervention [ISRCTN93896459]. Prev Med. 2010 Jul;51(1):31-6. PMID: 20380847. doi: 10.1016/j.ypmed.2010.03.015.
6. Whittemore R, Jeon S, Grey M. An internet obesity prevention program for adolescents. J Adolesc Health. 2013 Apr;52(4):439-47. PMID: 23299003. doi: 10.1016/j.jadohealth.2012.07.014.
7. Al-Eisa E, Al-Rushud A, Alghadir A, Anwer S, Al-Harbi B, Al-Sughaier N, et al. Effect of Motivation by "Instagram" on Adherence to Physical Activity among Female College Students. Biomed Res Int. 2016;2016:1546013. PMID: 27034927. doi: 10.1155/2016/1546013.
8. Ali HI, Attlee A, Alhebshi S, Elmi F, Al Dhaheri AS, Stojanovska L, et al. Feasibility Study of a Newly Developed Technology-Mediated Lifestyle Intervention for Overweight and Obese Young Adults. Nutrients. 2021 Jul 26;13(8). PMID: 34444707. doi: 10.3390/nu13082547.
9. Cavallo DN, Tate DF, Ries AV, Brown JD, DeVellis RF, Ammerman AS. A social media-based physical activity intervention: a randomized controlled trial. Am J Prev Med. 2012 Nov;43(5):527-32. PMID: 23079176. doi: 10.1016/j.amepre.2012.07.019.
10. Kattelmann KK, Bredbenner CB, White AA, Greene GW, Hoerr SL, Kidd T, et al. The effects of Young Adults Eating and Active for Health (YEAH): a theory-based Web-delivered intervention. J Nutr Educ Behav. 2014 Nov-Dec;46(6):S27-41. PMID: 25457733. doi: 10.1016/j.jneb.2014.08.007.
11. Melton BF, Buman MP, Vogel RL, Harris BS, Bigham LE. Wearable Devices to Improve Physical Activity and Sleep. Journal of Black Studies. 2016;47(6):610-25. doi: 10.1177/0021934716653349.
12. Memon A, Masood T, Awan W, Waqas A. The effectiveness of an incentivized physical activity programme (Active Student) among female medical students in Pakistan: A Randomized Controlled Trial. Journal of Pakistan Medical Association. 2018;68(10):1438-45.
