# Supplementary material for: Technology-Supported Physical Activity and Its Potential as a Tool to Promote Young Women’s Physical Activity and Physical Literacy: Systematic Review
Source: J Med Internet Res. 2024 Oct 18;26:e52302. doi: 10.2196/52302 (PMC11530733; doi:10.2196/52302)
Supplement: Multimedia Appendix 4 [file jmir_v26i1e52302_app4.pdf]

Multimedia Appendix 4. Intervention and result details for single sample studies

Author year, country	Sample	Other populations	Intervention	Intervention length	PA outcome	Data collection tool	Results	Effect size
**Adolescents (<19 years)**								
**Kerner, et al [1] 2019, UK**	N= 24 Age: 14-15 years – HI	Boys N= 38 Age: 14-15 years – HI	Students provided a Fitbit Charge HR	5 weeks, no follow-up	Days and minutes of MVPA	ActiGraph GT9X triaxial accelerometer	**Mean MVPA** Significant negative intervention effect Pre=202.44 ±32.53 vs. Post=190.85 (±35.06) $F (1, 26) = 6.90$, $p = 0.01$, $\eta^2 = 0.21$.	Significant ($\eta2=0.21$)
**Larsen et al [2] 2018, USA**	N= 21 Age: 14.7 years (SD 2.1) – IA and LA	No	The Niñas Saludables interactive website – PA tracking and goal setting tools. PA information and motivational content<br><br>Pedometer to track daily steps.<br><br>Initial in-person goal setting session with an RA	12 weeks, no follow-up	Minutes per week of MVPA<br><br>Accelerometer counts over 7 days	7DPAR ActiGraph GT3X+	**Mean weekly minutes of MVPA** Self-report: (24.7 ±26.1 vs. 79.4 ±46.8) P=<.001. Accelerometry: 24.8 ±38.3 vs. 10.4 ±30.2	Not reported
**Young adults (≥19 years)**								
**Curtis et al [3] 2020, Australia**	N= 16 Age: 23 (SD 3) years - HI	No	A weekly video of an exercise program developed by a clinical exercise physiologist.<br><br>Access to an Instagram page where the weekly video was posted along with additional PA and motivational material.<br><br>Phone calls from RAs to ensure participants had no technical issues	12 weeks, no follow-up	Minutes per week of VPA, MPA, walking and total MET	IPAQ- Short form	There were no significant intervention effects on any of the PA outcomes at 12-weeks. [interquartile range]: **VPA:** 0 [IQR -23-30] P=1.00 **MPA:** 60 [IQR -45, -170] P=0.43 **Walking:** -30 [IQR -265, -43], P=0.73 **Daily METS:** 297 [IQR =1197, -671], P=0.51	Not reported
**Joseph et al [4] 2015, USA**	N= 27 Age: 21.21 years (SD 2.29) years – OO and AA	No	Interactive website including PA tracking, goal setting and motivational tools. PA information and the ability to share progress.<br><br>In-person supervised PA sessions conducted for the first 3 months.	6 months, no follow-up	Minutes per week of MVPA	7DPAR	**Minutes of PA** **Baseline to midpoint (3-months)** 111.00 ±88.87 to 184.93 ±96.56 to 136.00 ±97.70)Wilcoxon Z = 2.05, P = 0.04<br><br>**Baseline to endpoint (6-months)** 136.00 ±97.70 Wilcoxon Z = 0.68, P = 0.50	Not reported
**Joseph et al [5] 2016, USA**	N= 31 Age: 21.3 years (SD 3.1) – OO and AA	No	Interactive website including PA tracking, goal setting and motivational tools. PA information and the ability to share progress.<br><br>In-person supervised PA sessions	3 months, no follow-up	Minutes per week of MVPA<br><br>Accelerometer counts over 7 days	7DPAR<br><br>ActiGraph GT3X+	**Minutes of MVPA** Self-reported: 99.4 ±103.8 vs. 134.7 ±119.5 P=0.15 Accelerometry: 45.0 ±51.6 vs. 29.8 ±37.28 P=0.08	Not reported

**Xian et al [6] 2017, USA and global***	N= 80<br>Age: 25 (range 21-29) years - HI	Males<br>N= 87<br>Age: 25 (range 21-29) years - HI	Pokémon GO players are encouraged to ambulate (e.g., walk, bike, or drive) to various physical locations in the real world to capture virtual creatures called Pokémon.	3 weeks, no follow-up	Time using Pokémon Go<br><br>Step count 3 weeks prior to downloading the game and 3 weeks after	Self-reported play time<br><br>Playtime recorded in the game<br><br>Steps recorded on iPhone Health app	**Total steps**<br>**Pre-intervention to post-intervention**<br>5446 ±2996 vs. 6569 ±3235 (95% CI 500- 1747)<br>P=0.001<br>10000 XP points = 2134 steps (95% CI, 1673–2595)<br>P=<0.001	Not reported

Notes: 7DPAR- Seven Day Physical Activity Recall, AA- African American, HI-Healthy individuals (not targeting any specific health concerns), IA- Insufficiently active, IPAQ- International Physical Activity Questionnaire, LA – Latina adolescents, MET- Metabolic equivalent of task, MPA- Moderate physical activity, MVPA- Moderate to vigorous physical activity, OO-Overweight or Obese, PA- Physical activity, RA- Research assistant, SD – Standard deviation, VPA- Vigorous physical activity * *JBI cross-sectional checklist used for this study as the retrospective observational design was better suited to this checklist, but it was included in the single sample analysis

References

1. Kerner C, Burrows A, McGrane B. Health wearables in adolescents: implications for body satisfaction, motivation and physical activity. International Journal of Health Promotion and Education. 2019;57(4):191-202. doi: 10.1080/14635240.2019.1581641.
2. Larsen B, Benitez T, Cano M, Dunsiger SS, Marcus BH, Mendoza-Vasconez A, et al. Web-Based Physical Activity Intervention for Latina Adolescents: Feasibility, Acceptability, and Potential Efficacy of the Ninas Saludables Study. J Med Internet Res. 2018 May 9;20(5):e170. PMID: 29743151. doi: 10.2196/jmir.9206.
3. Curtis RG, Ryan JC, Edney SM, Maher CA. Can Instagram be used to deliver an evidence-based exercise program for young women? A process evaluation. BMC Public Health. 2020 Oct 6;20(1):1506. PMID: 33023559. doi: 10.1186/s12889-020-09563-y.
4. Joseph RP, Dutton GR, Cherrington A, Fontaine K, Baskin M, Casazza K, et al. Feasibility, acceptability, and characteristics associated with adherence and completion of a culturally relevant internet-enhanced physical activity pilot intervention for overweight and obese young adult African American women enrolled in college. BMC Res Notes. 2015 Jun 2;8:209. PMID: 26032016. doi: 10.1186/s13104-015-1159-z.
5. Joseph RP, Pekmezi D, Dutton GR, Cherrington AL, Kim YI, Allison JJ, et al. Results of a Culturally Adapted Internet-Enhanced Physical Activity Pilot Intervention for Overweight and Obese Young Adult African American Women. J Transcult Nurs. 2016 Mar;27(2):136-46. PMID: 24934566. doi: 10.1177/1043659614539176.
6. Xian Y, Xu H, Xu H, Liang L, Hernandez AF, Wang TY, et al. An Initial Evaluation of the Impact of Pokemon GO on Physical Activity. J Am Heart Assoc. 2017 May 16;6(5). PMID: 28512111. doi: 10.1161/JAHA.116.005341.
