# Supplementary material for: Technology-Supported Physical Activity and Its Potential as a Tool to Promote Young Women’s Physical Activity and Physical Literacy: Systematic Review
Source: J Med Internet Res. 2024 Oct 18;26:e52302. doi: 10.2196/52302 (PMC11530733; doi:10.2196/52302)
Supplement: Multimedia Appendix 5 [file jmir_v26i1e52302_app5.pdf]

Multimedia Appendix 5. Findings from the cross-sectional studies

Author year, country	Sample	Other populations	Study aim	PA outcome	Data collection tool	Results	Technology use correlated with PA
**Adolescents (<19 years)**							
**Ng et al [1] 2021, Finland & Ireland**	N= 4128 Age: 11-15 years - HI	Males N= 3980 Age: 11-15 years - HI	Investigate the association between PAT ownership and self-reported PA and the potential influence of gender.	Self-reported days of 60+ minutes of MVPA	The F-SPA survey<br><br>CSPPA survey	**Days of MVPA**<br>Use of apps and wearable devices was significantly associated with MVPA (1.61 CI 95% 1.32 to 1.96 vs. 2.45 95% CI 2.00 to 3.02).<br><br>Greater association for wearable devices than mobile apps (30% vs. 22.9%) while non-users (19.2%) and non-owners (16.3%) reported less MVPA	Yes, with wearable devices being associated with higher levels of PA than mobile apps
**Young adults (≥19 years)**							
**McFadden [2] 2021, USA**	N= 289 Age: 19.6 years (SD 2.64) - HI	No	Analyse whether regular use of wearable exercise technology during PA was associated with increased PA	Days, minutes and intensity of PA<br><br>Use of wearable exercise technology	Online survey<br><br>Reported PA compared to ACSM PA guidelines	**Long-term users**<br>Users for > 6 months=27 (9%)<br>**ACSM PA guidelines**<br>Meeting = 23 (8%)<br>Long-term users most likely to be meeting ACSM PA guidelines	Yes, long-term users were the more likely to meet PA guidelines
**Nagata et al [3] 2021, USA**	N= 867 Age: 22.2 years (SD 2.0) - HI	Males N= 606 Gender diverse N= 10 Age: 22.2 years (SD 2.0) - HI	Look at three types of digital technology use and muscle building behaviours (exercise)	If participants increased their PA in the past year and if they used some form of digital technology to increase PA	The EAT 2018 survey	**Increased PA**<br>Weight-related self-monitoring apps: Prevalence ratio 1.48 (95% CI 1.32 to 1.66) P=0.001.	Yes, users of digital technology reported higher levels of PA
**Papalia et al [4] 2018, USA**	N= 581 Age: 21.07 years (SD 1.44) - HI	Males N= 494 Age: 21.07 years (SD 1.44) - HI	Examine the prevalence of technology-based PA and the potential relationship between device usage and overall PA.	Reported use of any PA devices<br><br>Minutes per week of VPA and MPA	Study specific survey<br><br>The GPAQ	**Weekly minutes of VPA**<br>Users 153.5 ±129.9 vs. non-users 105.9 ±142.1 P=< 0.001 t=-4.096<br>**Weekly minutes of MPA**<br>Users 157.7 ±132.0 vs. non-users 152.4 ±138.6 P=0.645 t=-0.462	Yes, but only for VPA
**Wang et al [5] 2019, China**	N= 779 Age: 20.3 years (SD 2.4) - HI	Males N= 466 Age: 20.3 years (SD 2.4) - HI	Investigate the impact current or previous use of technology-supported PA could have on overall PA engagement	Minutes of MPA, VPA, walking<br><br>Meeting WHO PA guidelines	IPAQ – simplified Chinese version	**Minutes of VPA**<br>Current users, previous users, and non-users: 90 vs. 25 vs. 0 chi-square= 49.42 (776) P=<.001<br>**Minutes of MPA**<br>Current users, previous users, and non-users: 90 vs. 30 vs. 20 chi-square= 27.45 (776) P=<.001.	Yes, for MPA, VPA and meeting WHO guidelines

						**Walking**<br>Current users, previous users, and non-users: 140 vs. 120 vs. 90 chi-square= 14.60 (776) P=0.88.<br>**Meeting WHO guidelines**<br>Current users, previous users, and non-users: 60% vs. 38.5% vs. 29.8% chi-square= 19.5 (776) P=<.001.	Previous users had higher levels of PA than non-users

Notes: ACSM- American College of Sports Medicine, CSPPA - Children's Sport Participation and Physical Activity, EAT - Eating and Activity over Time, F-SPA - Finnish School-aged Physical Activity, GPAQ- The Global Physical Activity Questionnaire, HI-Healthy individuals (not targeting any specific health concerns), PA- Physical activity, PAT- Physical activity tracker, IPAQ- International Physical Activity Questionnaire, MPA- Moderate physical activity, MVPA- Moderate to vigorous physical activity, SD – Standard deviation, VPA- Vigorous physical activity and WHO-World Health Organization

References

1. Ng K, Kokko S, Tammelin T, Kallio J, Belton S, O'Brien W, et al. Clusters of Adolescent Physical Activity Tracker Patterns and Their Associations With Physical Activity Behaviors in Finland and Ireland: Cross-Sectional Study. J Med Internet Res. 2020 Sep 1;22(9):e18509. PMID: 32667894. doi: 10.2196/18509.
2. McFadden C. Wearable Exercise Technology and the Impact on College Women’s Physical Activity. Quest. 2021;73(2):179-91. doi: 10.1080/00336297.2021.1891553.
3. Nagata JM, Hazzard VM, Ganson KT, Hahn SL, Neumark-Sztainer D, Eisenberg ME. Digital technology use and muscle-building behaviors in young adults. International Journal of Eating Disorders. 2021;55(2):207-14. doi: 10.1002/eat.23656.
4. Papalia Z, Wilson O, Bopp M, Duffey M. Technology-Based Physical Activity Self-Monitoring Among College Students. International journal of exercise science. 2018;11(7):1096-104.
5. Wang T, Ren M, Shen Y, Zhu X, Zhang X, Gao M, et al. The Association Among Social Support, Self-Efficacy, Use of Mobile Apps, and Physical Activity: Structural Equation Models With Mediating Effects. JMIR Mhealth Uhealth. 2019 Sep 25;7(9):e12606. PMID: 31573936. doi: 10.2196/12606.
