# Supplementary material for: Technology-Supported Physical Activity and Its Potential as a Tool to Promote Young Women’s Physical Activity and Physical Literacy: Systematic Review
Source: J Med Internet Res. 2024 Oct 18;26:e52302. doi: 10.2196/52302 (PMC11530733; doi:10.2196/52302)
Supplement: Multimedia Appendix 6 [file jmir_v26i1e52302_app6.pdf]

Multimedia Appendix 6. Summary of results in the expected direction by domain and element of physical literacy.

	Interactive website/social media			Physical activity tracking mobile app			Wearable fitness tracker			
	**Improvement [a]**	**Decline [a]**	**Null [a]**	**Improvement [a]**	**Decline [a]**	**Null [a]**	**Improvement [a]**	**Decline [a]**	**Null [a]**	**Reported positive outcomes**
**Psychological**										
Motivation	Al-Eisa et al [1] 2016[bc] ICG								Kerner et al [2] 2019 [d]	1/2
Engagement			Joseph et al [3] 2016 [d]							0/1
Enjoyment/ Confidence	Papalia et al [4] 2018[e]		Joseph et al [3] 2016 [d]							1/2
TOTAL	2	0	2	0	0	0	0	0	1	2/5 (40%) Code: ?
**Physical**										
Cardiovascular			Curtis et al [5] 2020 [d]							0/1
Endurance / Strength			Curtis et al [5] 2020 [d]							0/1
TOTAL	0	0	2	0	0	0	0	0	0	0/2 (0%) Code: 0
Overall impact on physical literacy										**2/7 (29%) Code: 0**

a Impact/association of study in the hypothesized direction

b Motivation not tested at baseline, but post-intervention participants reported the intervention was motivating.

c Intervention study with comparison groups

d Intervention study with a single sample

e Cross-sectional – shows correlation between technology and PA.

Note: The impact of interventions was marked as.

0 = no likely effect reported when ≤30% of the studies found changes in the expected direction (adapted from Page et al [6])

? = uncertain effect reported in 31-60% of the studies found changes in the expected direction (adapted from Page et al [6])

+ = positive effect reported when 61% to 100% of the studies found changes in the expected direction (adapted from Page et al [6])

References

1. Al-Eisa E, Al-Rushud A, Alghadir A, Anwer S, Al-Harbi B, Al-Sughaier N, et al. Effect of Motivation by "Instagram" on Adherence to Physical Activity among Female College Students. Biomed Res Int. 2016;2016:1546013. PMID: 27034927. doi: 10.1155/2016/1546013.
2. Kerner C, Burrows A, McGrane B. Health wearables in adolescents: implications for body satisfaction, motivation and physical activity. International Journal of Health Promotion and Education. 2019;57(4):191-202. doi: 10.1080/14635240.2019.1581641.
3. Joseph RP, Pekmezi D, Dutton GR, Cherrington AL, Kim YI, Allison JJ, et al. Results of a Culturally Adapted Internet-Enhanced Physical Activity Pilot Intervention for Overweight and Obese Young Adult African American Women. J Transcult Nurs. 2016 Mar;27(2):136-46. PMID: 24934566. doi: 10.1177/1043659614539176.
4. Papalia Z, Wilson O, Bopp M, Duffey M. Technology-Based Physical Activity Self-Monitoring Among College Students. International journal of exercise science. 2018;11(7):1096-104.
5. Curtis RG, Ryan JC, Edney SM, Maher CA. Can Instagram be used to deliver an evidence-based exercise program for young women? A process evaluation. BMC Public Health. 2020 Oct 6;20(1):1506. PMID: 33023559. doi: 10.1186/s12889-020-09563-y.
6. Page ZE, Barrington S, Edwards J, Barnett LM. Do active video games benefit the motor skill development of non-typically developing children and adolescents: A systematic review. J Sci Med Sport. 2017 Dec;20(12):1087-100. PMID: 28600111. doi: 10.1016/j.jsams.2017.05.001.
