# Supplementary material for: Technology-Supported Physical Activity and Its Potential as a Tool to Promote Young Women’s Physical Activity and Physical Literacy: Systematic Review
Source: J Med Internet Res. 2024 Oct 18;26:e52302. doi: 10.2196/52302 (PMC11530733; doi:10.2196/52302)
Supplement: Multimedia Appendix 7 [file jmir_v26i1e52302_app7.pdf]

Multimedia Appendix 7. Quality assessment for each study design.

RCT

Question	Cavallo et al [1] 2012	Dzielska et al [2] 2020	Kattelmann et al [3] 2014	Melton et al [4] 2016	Memon et al [5] 2017	Ridgers et al [6] 2021	Seah and Koh [7] 2021	Slootmaker et al [8] 2010	Whittemore et al [9] 2013
Was true randomization used for assignment of participants to treatment groups?	✓	✓	✓	✓	✓	✓	✓	✓	✓
Was allocation to treatment groups concealed?	NR	NR	NR	NR	✓	NR	NR	✓	NR
Were treatment groups similar at the baseline?	X	✓	✓	✓	X	✓	NR	✓	✓
Were participants blind to treatment assignment?	✓	✓	X	✓	NR	NR	NR	NR	NR
Were those delivering treatment blind to treatment assignment?	NA	NA	NA	NA	NA	X	X	NR	NR
Were outcomes assessors blind to treatment assignment?	NA	NR	NA	NA	NR	X	NA	NA	NR
Were treatment groups treated identically other than the intervention of interest?	✓	✓	✓	✓	✓	✓	✓	✓	✓
Was follow-up complete and if not, were differences between groups in terms of their follow-up adequately described and analyzed?	✓	X	X	X	NR	✓	X	✓	✓
Were participants analyzed in the groups to which they were randomized?	✓	✓	✓	NR	✓	✓	✓	✓	✓
Were outcomes measured in the same way for treatment groups?	✓	✓	✓	✓	✓	✓	✓	✓	✓
Were outcomes measured in a reliable way?	NR	X	✓	✓	X	✓	✓	X	X
Was appropriate statistical analysis used?	✓	X	X	✓	X	✓	✓	✓	✓
Was the trial design appropriate, and any deviations from the standard RCT design accounted for in the conduct and analysis of the trial?	✓	✓	✓	✓	✓	✓	X	✓	✓

Quasi-experimental

Question	Al-Eisa et al [10] 2016	Ali et al [11] 2021	Curtis et al [12] 2020	Glaser et al [13] 2024	Joseph et al [14] 2015	Joseph et al [15] 2016	Kerner et al [16] 2019	Larsen et al [17] 2018
Is it clear in the study what is the "cause" and what is the ""effect"" (ie, there is no confusion about which variable comes first)?	✓	✓	✓	✓	✓	✓	✓	✓
Were the participants included in any comparisons similar?	NR	NR	NA	✓	NA	NA	NA	NA
Were the participants included in any comparisons receiving similar treatment or care, other than the exposure or intervention of interest?	NR	✓	NR	NR	✓	✓	NR	NR
Was there a control group?	✓	✓	NA	✓	NA	NA	NA	NA
Were there multiple measurements of the outcome both pre and post the intervention or exposure?	X	X	✓	✓	✓	X	X	X
Was follow-up complete and if not, were differences between groups in terms of their follow-up adequately described and analyzed?	X	X	NR	X	✓	X	X	✓
Were the outcomes of participants included in any comparisons measured in the same way?	✓	✓	NA	✓	NA	NA	NA	NA
Were outcomes measured in a reliable way?	X	X	✓	X	✓	✓	✓	X
Was appropriate statistical analysis used?	X	X	X	X	X	X	X	X

Cross-sectional/retrospective observational

Question	McFadden [18] 2021	Nagata et al [19] 2021	Ng et al [20] 2020	Papalia et al [21] 2018	Wang et al [22] 2019	Xian et al [23] 2017*
Were the criteria for inclusion in the sample clearly defined?	NR	X	✓	X	✓	✓
Were the study subjects and the setting described in detail?	✓	✓	✓	✓	✓	✓
Was the exposure measured in a valid and reliable way?	✓	✓	✓	✓	✓	X
Were objective, standard criteria used for measurement of the condition?	NR	NR	✓	NR	✓	✓
Were confounding factors identified?	NR	✓	✓	NR	✓	NR
Were strategies to deal with confounding factors stated?	NR	✓	✓	NR	✓	NR
Were the outcomes measured in a valid and reliable way?	✓	✓	✓	✓	✓	✓
Was appropriate statistical analysis used?	✓	✓	✓	✓	✓	✓

✓	Positive response to question
X	Negative response to question
NR	Not reported (which was considered a negative response)
NA	NA = not applicable (which was not considered on the percentage calculation)

*JBI cross-sectional checklist used for this study as the retrospective observational design was better suited to this checklist

References

1. Cavallo DN, Tate DF, Ries AV, Brown JD, DeVellis RF, Ammerman AS. A social media-based physical activity intervention: a randomized controlled trial. Am J Prev Med. 2012 Nov;43(5):527-32. PMID: 23079176. doi: 10.1016/j.amepre.2012.07.019.
2. Dzielska A, Mazur J, Nalecz H, Oblacinska A, Fijalkowska A. Importance of Self-Efficacy in Eating Behavior and Physical Activity Change of Overweight and Non-Overweight Adolescent Girls Participating in Healthy Me: A Lifestyle Intervention with Mobile Technology. Nutrients. 2020 Jul 17;12(7). PMID: 32709005. doi: 10.3390/nu12072128.
3. Kattelmann KK, Bredbenner CB, White AA, Greene GW, Hoerr SL, Kidd T, et al. The effects of Young Adults Eating and Active for Health (YEAH): a theory-based Web-delivered intervention. J Nutr Educ Behav. 2014 Nov-Dec;46(6):S27-41. PMID: 25457733. doi: 10.1016/j.jneb.2014.08.007.
4. Melton BF, Buman MP, Vogel RL, Harris BS, Bigham LE. Wearable Devices to Improve Physical Activity and Sleep. Journal of Black Studies. 2016;47(6):610-25. doi: 10.1177/0021934716653349.
5. Memon A, Masood T, Awan W, Waqas A. The effectiveness of an incentivized physical activity programme (Active Student) among female medical students in Pakistan: A Randomized Controlled Trial. Journal of Pakistan Medical Association. 2018;68(10):1438-45.
6. Ridgers ND, Timperio A, Ball K, Lai SK, Brown H, Macfarlane S, et al. Effect of commercial wearables and digital behaviour change resources on the physical activity of adolescents attending schools in socio-economically disadvantaged areas: the RAW-PA cluster-randomised controlled trial. Int J Behav Nutr Phys Act. 2021 Apr 12;18(1):52. PMID: 33845853. doi: 10.1186/s12966-021-01110-1.
7. Seah MLC, Koh KT. The efficacy of using mobile applications in changing adolescent girls' physical activity behaviour during weekends. European Physical Education Review. 2020;27(1):113-31. doi: 10.1177/1356336x20930741.
8. Slootmaker SM, Chinapaw MJ, Seidell JC, van Mechelen W, Schuit AJ. Accelerometers and Internet for physical activity promotion in youth? Feasibility and effectiveness of a minimal intervention [ISRCTN93896459]. Prev Med. 2010 Jul;51(1):31-6. PMID: 20380847. doi: 10.1016/j.ypmed.2010.03.015.
9. Whittemore R, Jeon S, Grey M. An internet obesity prevention program for adolescents. J Adolesc Health. 2013 Apr;52(4):439-47. PMID: 23299003. doi: 10.1016/j.jadohealth.2012.07.014.
10. Al-Eisa E, Al-Rushud A, Alghadir A, Anwer S, Al-Harbi B, Al-Sughaier N, et al. Effect of Motivation by "Instagram" on Adherence to Physical Activity among Female College Students. Biomed Res Int. 2016;2016:1546013. PMID: 27034927. doi: 10.1155/2016/1546013.

11. Ali HI, Attlee A, Alhebshi S, Elmi F, Al Dhaheri AS, Stojanovska L, et al. Feasibility Study of a Newly Developed Technology-Mediated Lifestyle Intervention for Overweight and Obese Young Adults. Nutrients. 2021 Jul 26;13(8). PMID: 34444707. doi: 10.3390/nu13082547.

12. Curtis RG, Ryan JC, Edney SM, Maher CA. Can Instagram be used to deliver an evidence-based exercise program for young women? A process evaluation. BMC Public Health. 2020 Oct 6;20(1):1506. PMID: 33023559. doi: 10.1186/s12889-020-09563-y.

13. Glaser M, Green G, Barak S, Bord S, Levi S, Jakobovich R, et al. The effects of the Friendship Online Intervention Program on physical activity, substance abuse, psychosomatic symptoms, and well-being among at-risk youth. J Adolesc. 2024 Feb;96(2):251-65. PMID: 37985148. doi: 10.1002/jad.12272.

14. Joseph RP, Dutton GR, Cherrington A, Fontaine K, Baskin M, Casazza K, et al. Feasibility, acceptability, and characteristics associated with adherence and completion of a culturally relevant internet-enhanced physical activity pilot intervention for overweight and obese young adult African American women enrolled in college. BMC Res Notes. 2015 Jun 2;8:209. PMID: 26032016. doi: 10.1186/s13104-015-1159-z.

15. Joseph RP, Pekmezi D, Dutton GR, Cherrington AL, Kim YI, Allison JJ, et al. Results of a Culturally Adapted Internet-Enhanced Physical Activity Pilot Intervention for Overweight and Obese Young Adult African American Women. J Transcult Nurs. 2016 Mar;27(2):136-46. PMID: 24934566. doi: 10.1177/1043659614539176.

16. Kerner C, Burrows A, McGrane B. Health wearables in adolescents: implications for body satisfaction, motivation and physical activity. International Journal of Health Promotion and Education. 2019;57(4):191-202. doi: 10.1080/14635240.2019.1581641.

17. Larsen B, Benitez T, Cano M, Dunsiger SS, Marcus BH, Mendoza-Vasconez A, et al. Web-Based Physical Activity Intervention for Latina Adolescents: Feasibility, Acceptability, and Potential Efficacy of the Ninas Saludables Study. J Med Internet Res. 2018 May 9;20(5):e170. PMID: 29743151. doi: 10.2196/jmir.9206.

18. McFadden C. Wearable Exercise Technology and the Impact on College Women’s Physical Activity. Quest. 2021;73(2):179-91. doi: 10.1080/00336297.2021.1891553.

19. Nagata JM, Hazzard VM, Ganson KT, Hahn SL, Neumark-Sztainer D, Eisenberg ME. Digital technology use and muscle-building behaviors in young adults. International Journal of Eating Disorders. 2021;55(2):207-14. doi: 10.1002/eat.23656.

20. Ng K, Kokko S, Tammelin T, Kallio J, Belton S, O'Brien W, et al. Clusters of Adolescent Physical Activity Tracker Patterns and Their Associations With Physical Activity Behaviors in Finland and Ireland: Cross-Sectional Study. J Med Internet Res. 2020 Sep 1;22(9):e18509. PMID: 32667894. doi: 10.2196/18509.

21. Papalia Z, Wilson O, Bopp M, Duffey M. Technology-Based Physical Activity Self-Monitoring Among College Students. International journal of exercise science. 2018;11(7):1096-104.

22. Wang T, Ren M, Shen Y, Zhu X, Zhang X, Gao M, et al. The Association Among Social Support, Self-Efficacy, Use of Mobile Apps, and Physical Activity: Structural Equation Models With Mediating Effects. JMIR Mhealth Uhealth. 2019 Sep 25;7(9):e12606. PMID: 31573936. doi: 10.2196/12606.
23. Xian Y, Xu H, Xu H, Liang L, Hernandez AF, Wang TY, et al. An Initial Evaluation of the Impact of Pokemon GO on Physical Activity. J Am Heart Assoc. 2017 May 16;6(5). PMID: 28512111. doi: 10.1161/JAHA.116.005341.
